# Discovery of Coumarin as Microtubule Affinity-Regulating Kinase 4 Inhibitor That Sensitize Hepatocellular Carcinoma to Paclitaxel

**DOI:** 10.3389/fchem.2019.00366

**Published:** 2019-05-24

**Authors:** Xianyan Shen, Xuesha Liu, Shunli Wan, Xin Fan, Huaiyu He, Rong Wei, Wenchen Pu, Yong Peng, Chun Wang

**Affiliations:** ^1^Chengdu Institute of Biology, Chinese Academy of Sciences, Chengdu, China; ^2^State Key Laboratory of Biotherapy and Cancer Center, West China Hospital and College of Life Sciences, Sichuan University and Collaborative Innovation Center of Biotherapy, Chengdu, China

**Keywords:** MARK4, paclitaxel, Hepatocellular carcinoma, drug resistance, inhibitor

## Abstract

Hepatocellular carcinoma (HCC) is one of the most prevalent cancers worldwide. Nowadays, pharmacological therapy for HCC is in urgent needs. Paclitaxel is an effective drug against diverse solid tumors, but commonly resisted in HCC patients. We recently have disclosed that microtubule affinity-regulating kinase 4 (MARK4) increases the microtubule dynamics and confers paclitaxel resistance in HCC, suggesting MARK4 as an attractive target to overcome paclitaxel resistance. Herein, we synthesized and identified coumarin derivatives **50** as a novel MARK4 inhibitor. Biological evaluation indicated compound **50** directly interacted with MARK4 and inhibited its activity *in vitro*, suppressed cell viability and induced apoptosis of HCC cells in a MARK4-dependent manner. Importantly, compound **50** significantly increased the drug response of paclitaxel treatment to HCC cells, providing a promise strategy to HCC treatment and broadening the application of paclitaxel in cancer therapy.

**Graphical Abstract:**
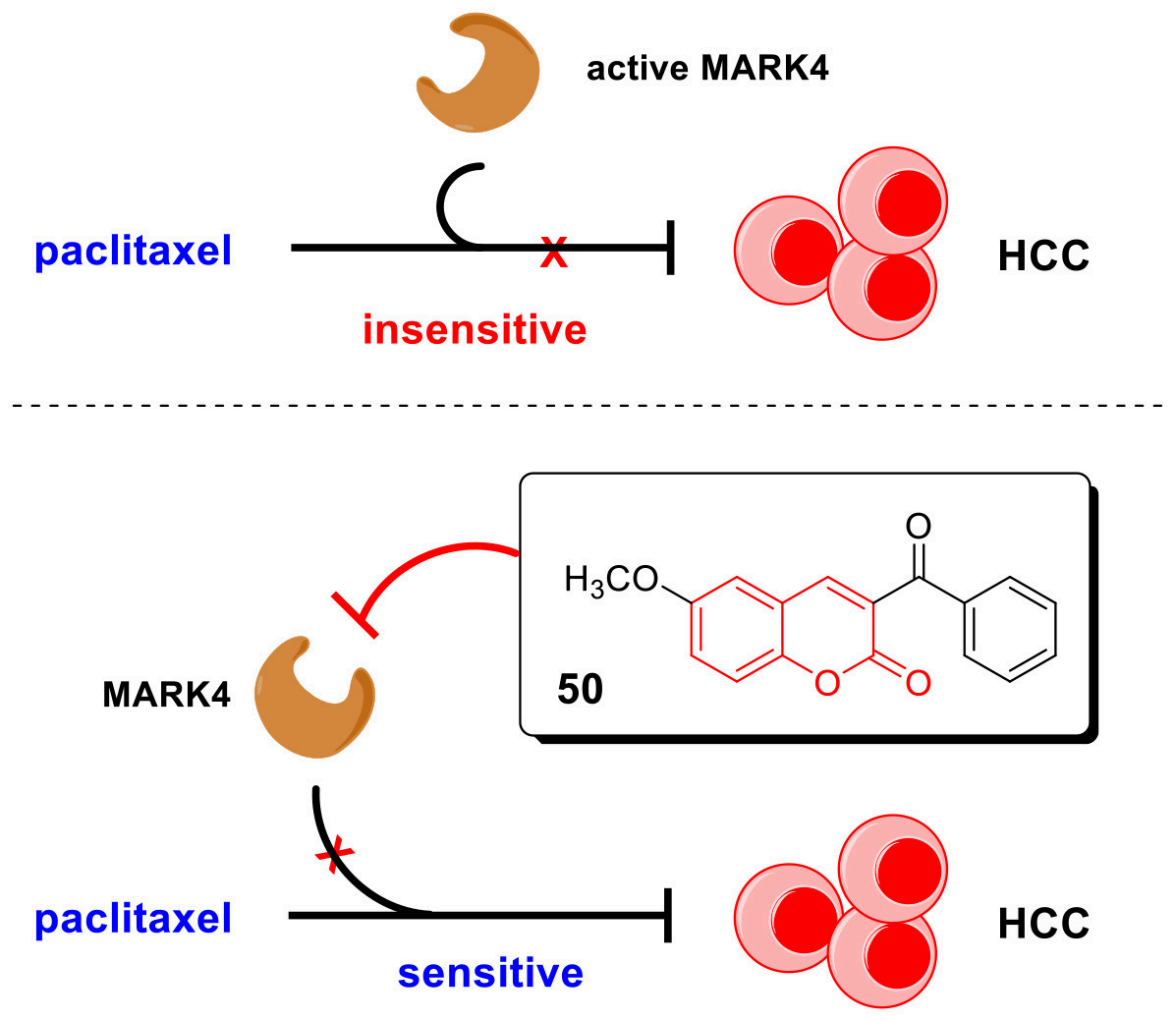
In this work, we discovered coumarin derivatives **50** as a novel MARK4 inhibitor. Compound **50** directly interacted with MARK4 and inhibited its activity *in vitro*, significantly suppressed cell viability and induced apoptosis of HCC cells. Importantly, compound **50** could sensitize HCC cells to paclitaxel treatment, providing a potential strategy to HCC treatment.

## Introduction

Hepatocellular carcinoma (HCC) is one of the leading causes of human death worldwide (Bray et al., 2018). Although several therapies have been developed, such as sorafenib, the first-line targeted drug for advanced HCC, their efficacies are not satisfactory, rendering a poor prognosis for HCC patients (Galle, 2008; Llovet et al., 2008). Novel therapy is urgently needed for HCC treatment. Paclitaxel, firstly isolated in 1971 from the Pacific yew, is the Food and Drug Administration (FDA)-approved therapy exhibiting promising efficacy against diverse solid tumors including breast, ovarian, lung, cervical and pancreatic cancers (Wang et al., 2011; Pignata et al., 2015; Schmid et al., 2018). Mechanically, paclitaxel stabilizes the microtubule polymer and protects chromosomes from achieving a metaphase spindle configuration, blocking the mitosis progression and triggering apoptosis or cell cycle arrest without cell division (Bharadwaj and Yu, 2004; Jordan and Wilson, 2004; Brito et al., 2008). However, the data of clinical trial reveals that the anticancer effect of paclitaxel toward HCC patients is limited (Chao et al., 1998; Kavallaris, 2010). Our recent work indicates the depletion of microRNA-122 (miR-122, a dominant microRNA in normal liver cells) (Bandiera et al., 2015) upregulates septin-9 expression, facilitates the phosphorylation of microtubule-associated proteins 4 (MAP4) by microtubule affinity-regulating kinase (MARK4), causes detachment of the MAP4 from the microtubule, increasing microtubule dynamics and therefore conferring paclitaxel resistance in HCC (Sun et al., 2016). In this process, MARK4-mediated MAP4 phosphorylation is a key step.

MARK4 belongs to the family of serine/threonine kinases, representing a subfamily of calcium/calmodulin-dependent protein kinase (Drewes et al., 1997; Goodwin et al., 2014; Rovina et al., 2014) MARK4 has 752 amino acid residues in length and consists of a catalytic domain, a ubiquitin-associated domain and a kinase domain in structure (Kato et al., 2001; Matenia and Mandelkow, 2009). MARK4 is up-regulated in multiple human malignancies, including glioma, metastatic breast carcinoma and HCC (Kato et al., 2001; Beghini et al., 2003; Heidary Arash et al., 2017), and promotes tumor progression and development by participating oncogenic signaling pathways (Heidary Arash et al., 2017). Unlike other MARK members (MARK1-3), MARK4 is unique in its ability to exhibit direct association with microtubule (Trinczek et al., 2004), making MARK4 an attractive target to sensitize HCC to paclitaxel treatment.

Increasing endeavor have been devoted to the development of small-molecule MARK4 inhibitors. Before the clarification of MARK4 crystal structure, combined molecular dynamic simulation and pharmacophore-based virtual screening identified six 9-oxo-9H-acridin-10-yl derivatives to prevent MARK4 activity (Jenardhanan et al., 2014). In 2016, the crystal structure of MARK4 catalytic domain in complex with a pyrazolopyrimidine was disclosed (Sack et al., 2016), and thereafter a series of bioactive coumarins or flavonoids from ZINC database was discovered as potential MARK4 inhibitors via structure-based virtual high-throughput screening (Mohammad et al., 2018). Until now, scientists have also recruited polyphenolics, pyrrolopyrimidinone, isatin-triazole hydrazone and 2-heteroarylchromone derivatives into the arsenal for MARK4 inhibition (Katz et al., 2017; Khan et al., 2017; Parveen et al., 2018; Aneja et al., 2019). However, the development of MARK4 inhibitor is still in its infancy due to unfavorable potency and selectivity. Importantly, whether MARK4 inhibitor could sensitize HCC to paclitaxel treatment remains unknown.

In this work, we discovered compound **50** as a novel MARK4 inhibitor via computer-aided virtual screening. Compound **50** associated with MARK4 catalytic domain and inhibited MARK4 activity *in vitro* with an IC_50_ value of 1.301 μM. In HCC cells, compound **50** suppressed cell proliferation in a MARK4-dependent manner. Moreover, compound **50** could sensitize the anticancer function of paclitaxel against HCC cells, providing a new therapeutic approach for HCC and enlarging the potential application of paclitaxel in cancer treatment.

## Results and Discussion

**Figure 1:**
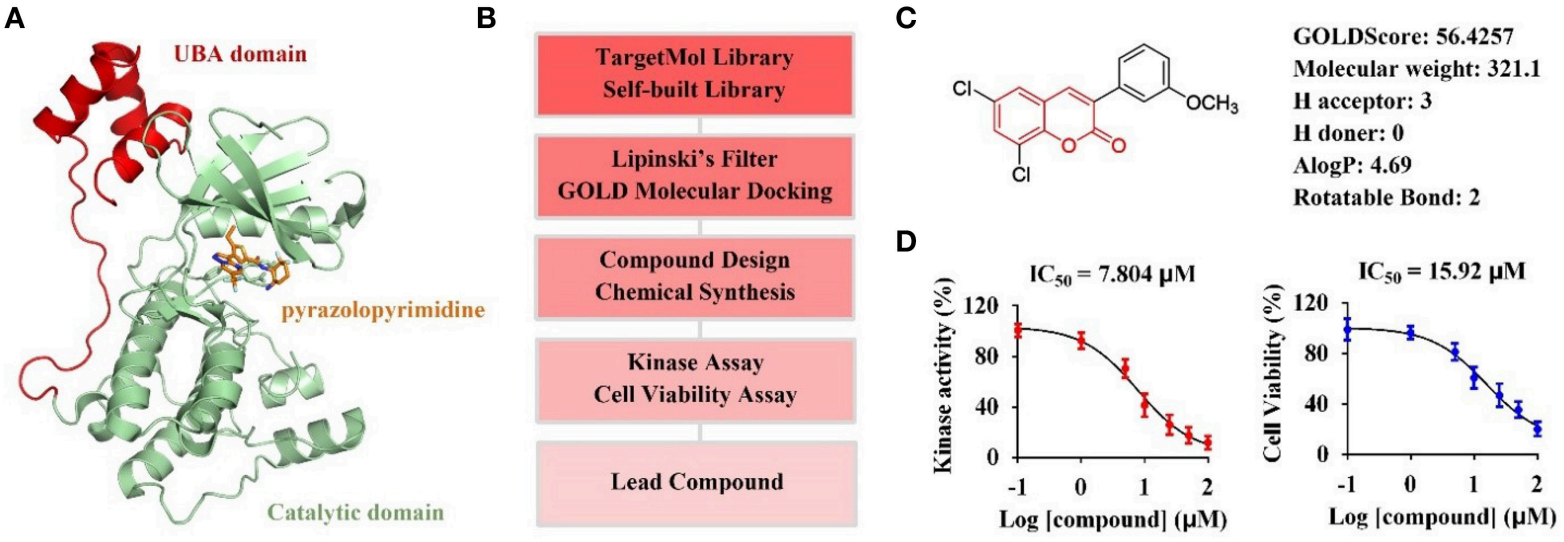
Computer-aided virtual screening of potential MARK4 inhibitors. **(A)** Crystal structure of MARK4 catalytic domain in complex with pyrazolopyrimidine inhibitor (PDB ID: 5ES1). **(B)** Workflow for computer-aided screening of MARK4 inhibitor. **(C)** Selected hit compound with coumarin moiety after Lipinski's filtering and GOLD molecular docking. **(D)** Biological evaluation of hit coumarin via kinase assay (left) using MARK4 as enzyme and cell viability assay (right) in HepG2 cells.

The catalytic domain of MARK4 recognizes its substrate MAP4, resulting in the phosphorylation of MAP4 to increase microtubule dynamics, is a key motif for MARK4 function (Trinczek et al., 2004). Recently, the crystal structure of MARK4 in complex with its inhibitor (PDB ID: 5ES1) have been disclosed (Figure 1A), facilating the discovery of small-molecule MARK4 inhibitors (Sack et al., 2016). Thus, we planned to establish the molecular docking model based on this crystal structure, and conduct computer-aided virtual screening of TargetMol and self-built compound library via Lipinski's filtering and GOLD molecular docking in Discovery Studio v3.1 software; The hit compounds will be synthesized and submitted to biological evaluation to obtain promising lead compounds (Figure 1B).

Small molecules after Lipinski's filtering in a library containing 5,972 compounds were screened *in silico* through GOLD molecular docking. Higher GoldScore.Fitness value implies higher potential affinity between protein and small molecules. Among the hit compounds, 3-arylcoumarin 6,8-dichloro-3-(3-methoxyphenyl)-2H-chromen-2-one had favorable drug-likeness and GOLDScore (Figure 1C). This coumarin was then submitted to kinase assay and cell viability assay to evaluate its biological activity. The results suggested 6,8-dichloro-3-(3-methoxyphenyl)-2H-chromen-2-one inhibited MARK4 activity with an IC_50_ value of 7.804 μM and suppressed the cell viability of HepG2 cells with an IC_50_ value of 15.92 μM (Figure 1D). Thus, we speculated that coumarin derivatives were favorable to inhibit MARK4 function.

**Scheme 1:**
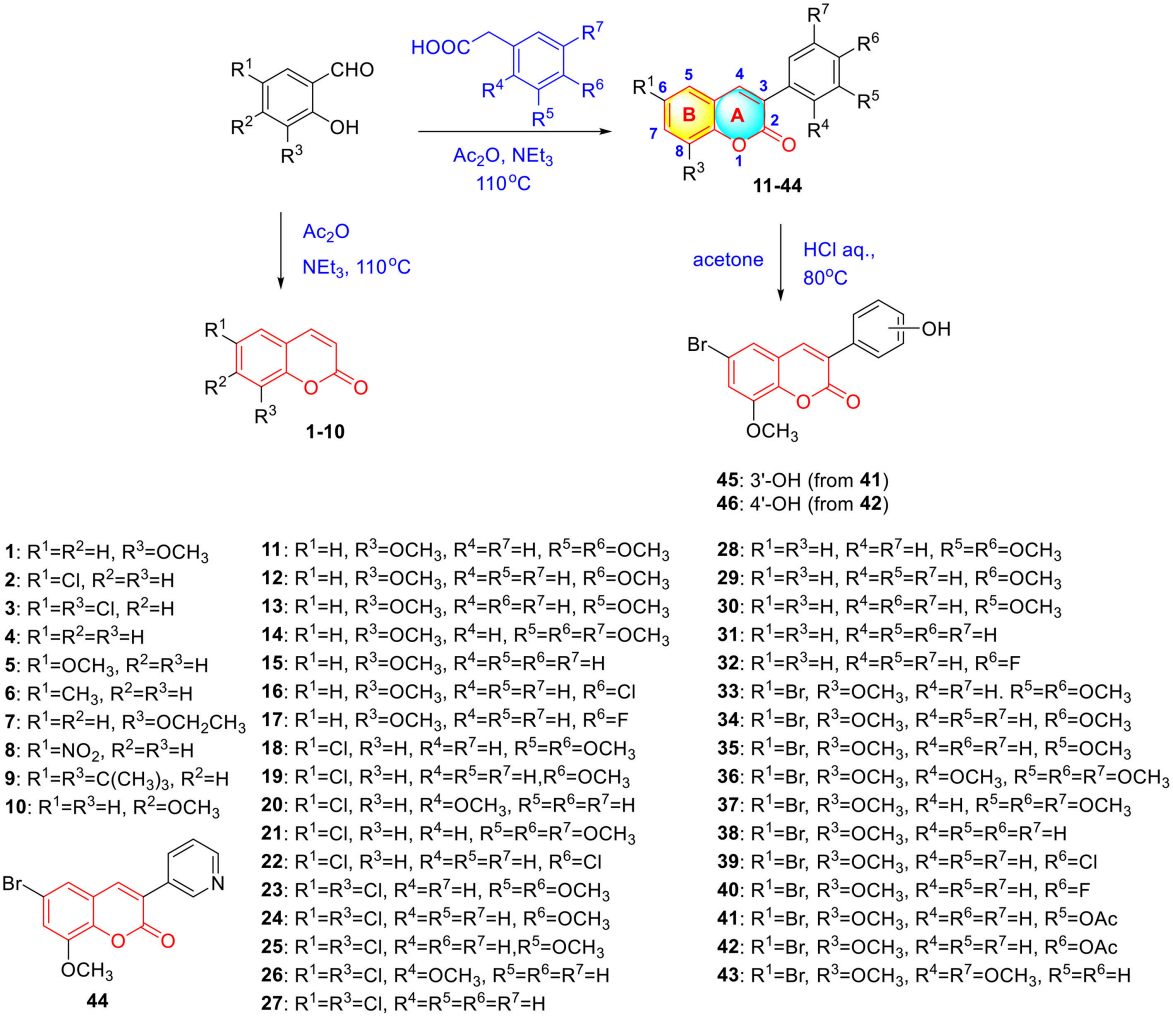
Synthesis of coumarins **1**–**46** via Perkin reaction.

**Scheme 2:**
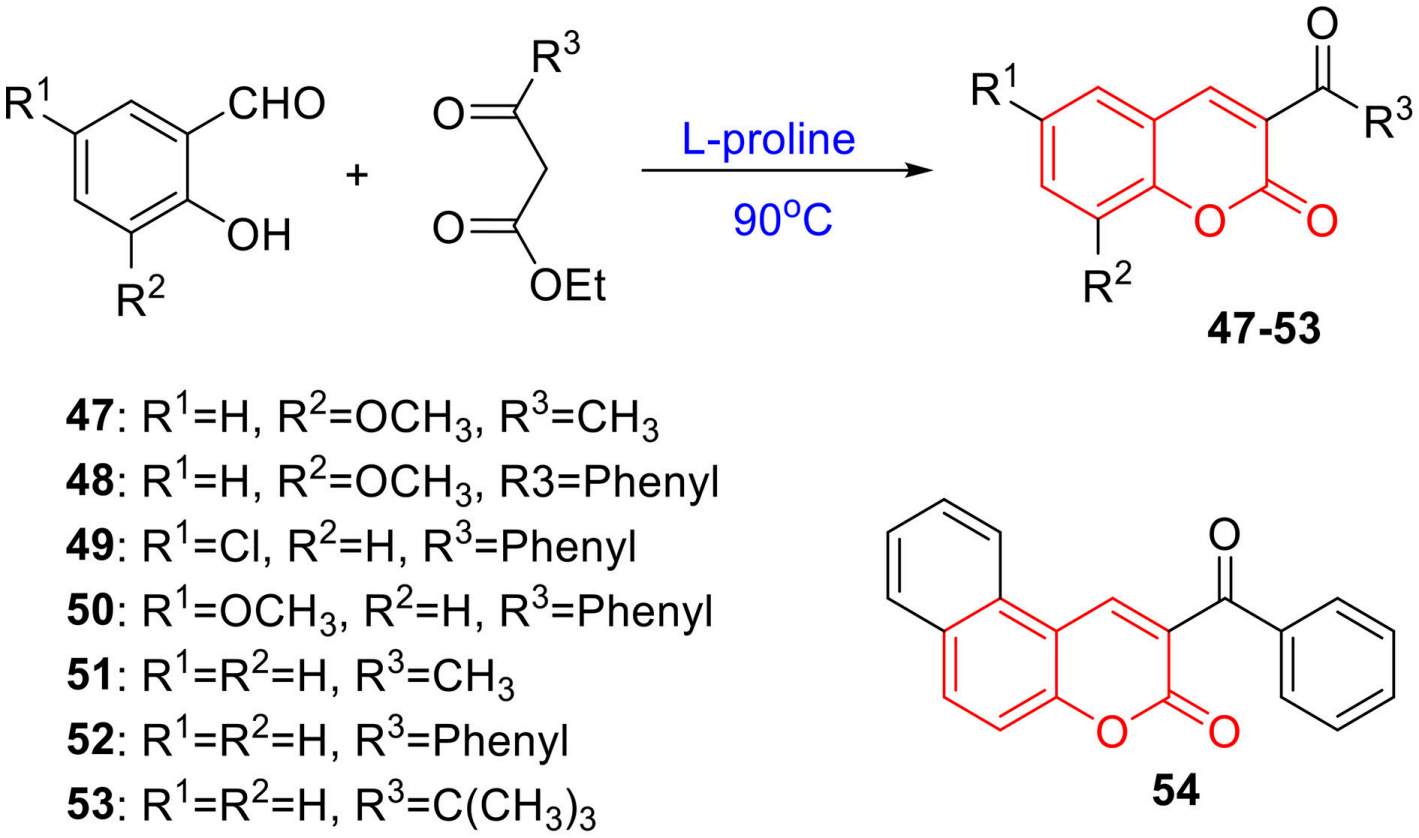
Synthesis of coumarins **47**–**54** via Knoevenagel reaction.

To verify this speculation, a series of coumarin derivatives, including 3-acry-, 3-aryl- 4-alkyl-, or 4-aryl coumarins and 3-arylthiocoumarins, were designed and synthesized in vision with structural and electronic features. Starting from substituted salicylaldehydes, coumarins **1–10** were successfully prepared via the Perkin reaction (Scheme 1). 3-Arylcoumarins **11–46** were synthesized from salicylaldehyde derivatives and phenylacetic acid derivatives through the Perkin condensation followed by acid-promoted hydrolysis if necessary, which were described in our previous work (Scheme 1) (Pu et al., 2014a). Moreover, salicylaldehydes and reactive methylene compounds were utilized as substrates in the presence of L-proline via the Knoevenagel reaction (Karade et al., 2008), 3-acrycoumarin **47–54** were afforded with high yields (Scheme 2).

**Scheme 3:**
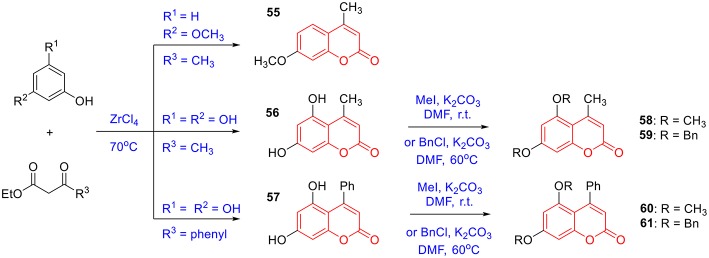
Synthesis of coumarins **55**–**61** based on the Pechmann reaction.

**Scheme 4:**
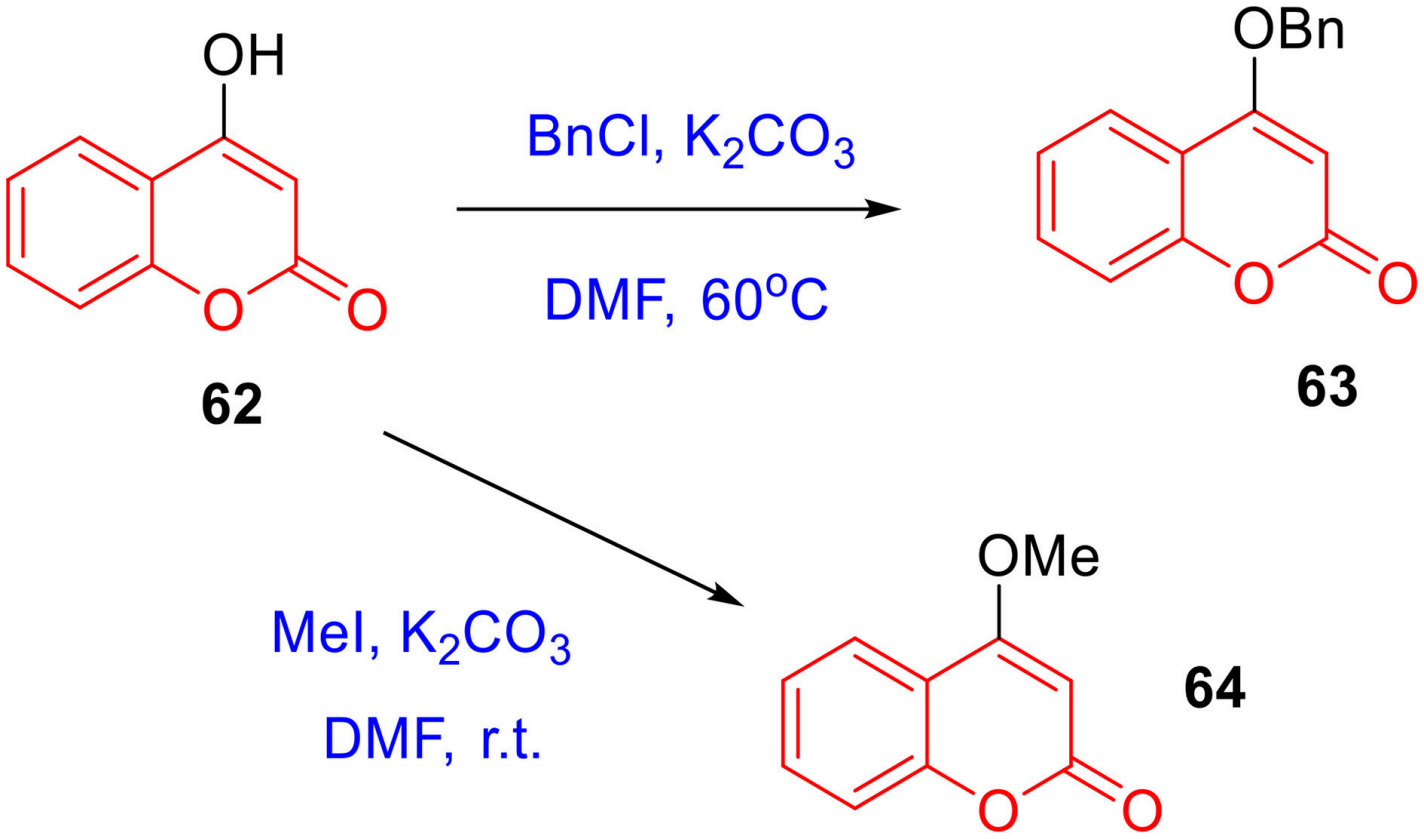
Preparation of **63** and **64** from alkylation of **62**.

**Scheme 5:**
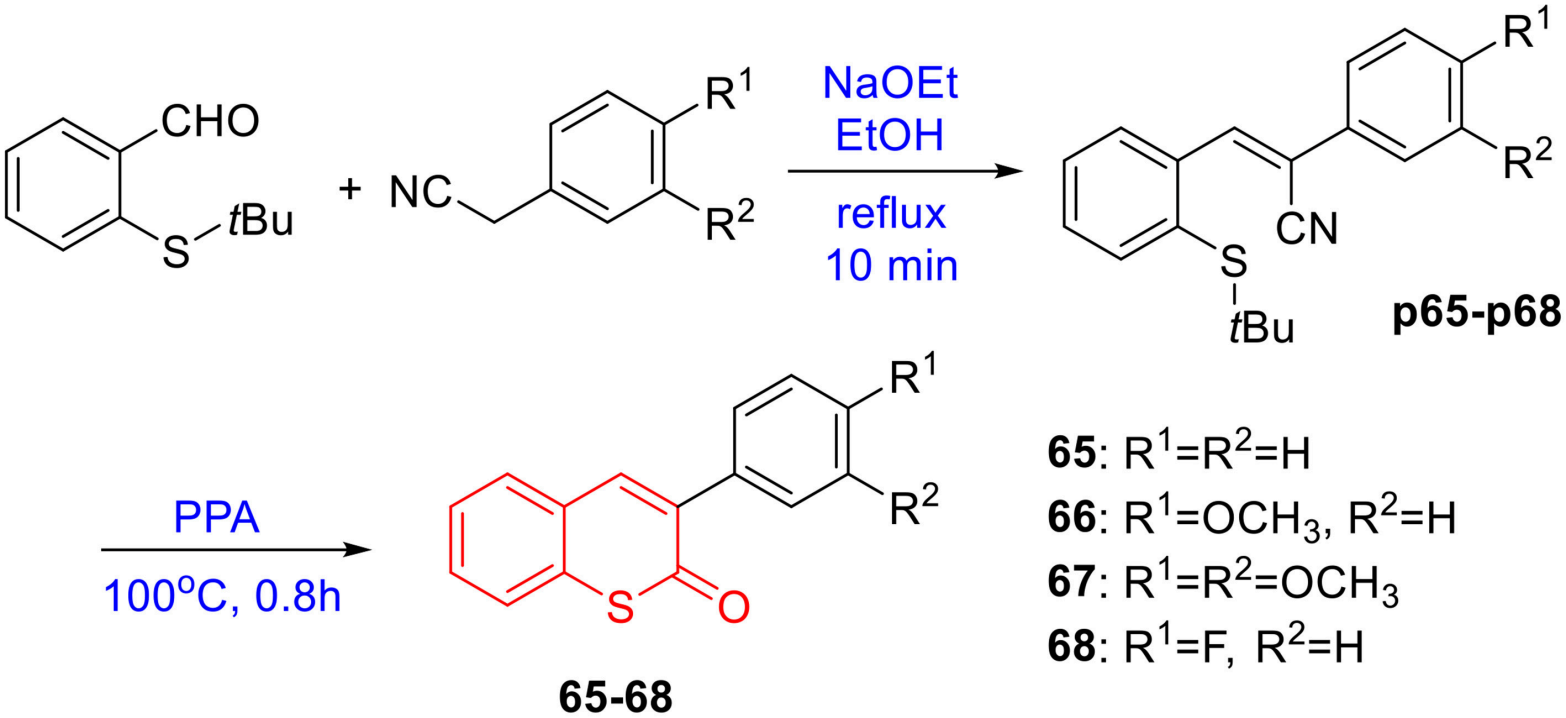
Synthesis of thiocoumarin **65**–**68**.

To prepare 4-methyl or 4-phenyl coumarins, we adapted Pechmann reaction-based strategy (Smitha and Sanjeeva Reddy, 2004). By using phenol derivatives and reactive methylene compounds as substrates, zircomiun tetrachloride as the mediator, compound **55–57** were synthesized with acceptable yields (Scheme 3). **56** and **57** were subsequently transformed into **58–61** via alkylation (Scheme 3). Similarly, 4-benzyloxy- or 4-methoxylcoumarins (**63**, **64**) were obtained from commercially-available compound **62** through benzylation and methylation, respectively (Scheme 4). In addition, following a two-step strategy (Meth-Cohn and Tarnowski, 1978), we also synthesized thiocoumarin **65–68** with moderate overall yields (Scheme 5). Together, through multiple synthetic strategies, sixty-eight coumarin derivatives were prepared as candidates for the investigation of potential MARK4 inhibitors.

**Figure 2:**
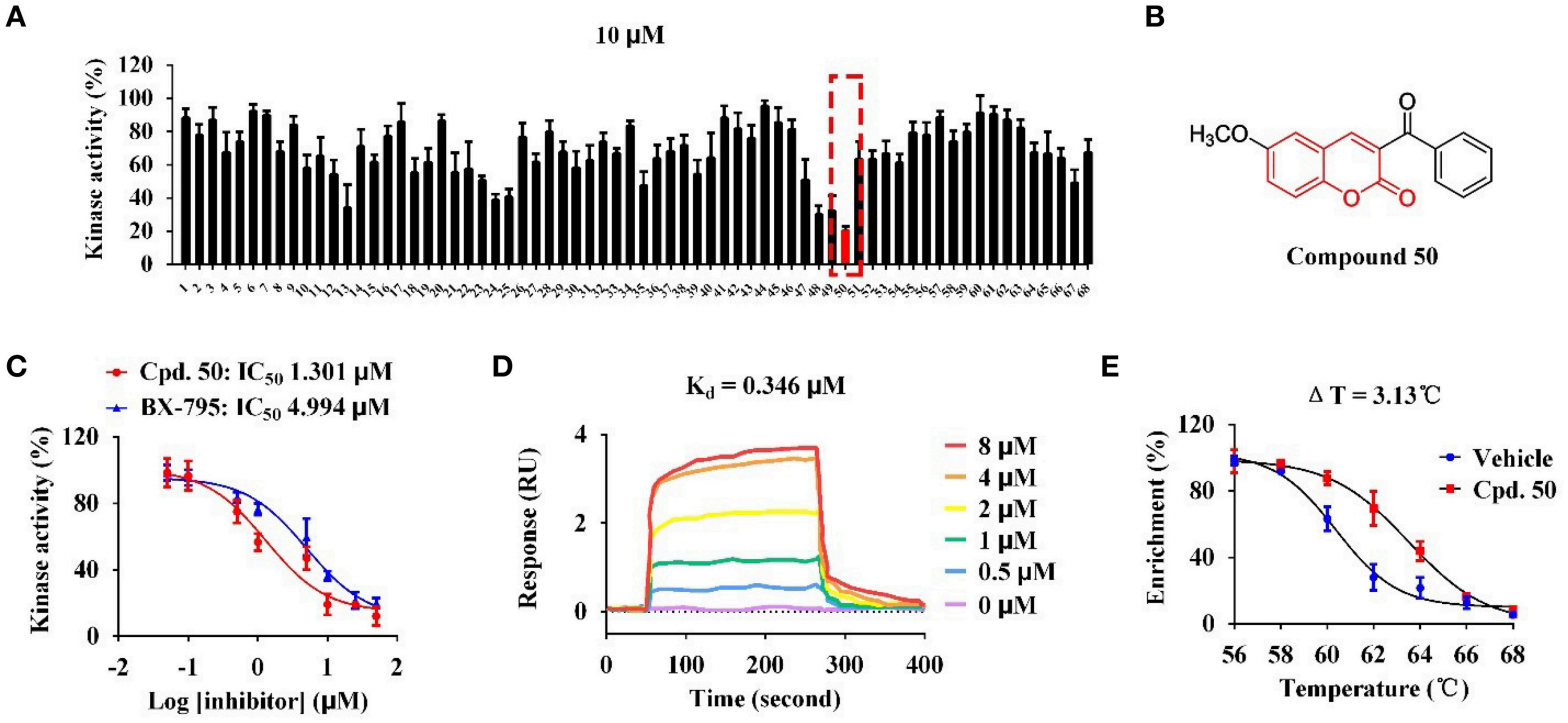
Compound **50** targeted catalytic domain to inhibit MARK4 activity *in vitro*. **(A)** Kinase assay of coumarin derivatives at the concentration of 10 μM using MARK4 as enzyme. **(B)** Chemical structure of compound **50**. **(C)** Kinase assay of compound 50 and BX-795 at varied concentrations using MARK4 as enzyme. **(D)** Surface plasmon resonance (SPR) assay of compound **50**. **(E)** Thermal shift assay of compound **50**.

With these coumarins in hand, we performed kinase assay using MARK4 catalytic domain as enzyme to test the MARK4 inhibitory activity of these samples. As in Figure 2A and Table 1, 3-benzoyl-6-methoxy-2*H*-chromen-2-one (**50**, Figure 2B) was the most potent MARK4 inhibitor with about 80% of MARK4 inhibition at 10 μM and had an IC_50_ value of 1.301 μM for its anti-MARK4 function (Figure 2C). By contrast, BX-795, an ATP-competitive MARK4 inhibitor (Feldman et al., 2005), had a reduced inhibitory activity against MARK4 with an IC_50_ value of 4.994 μM (Figure 2C). To characterize the selectivity of compound **50**, we performed kinase activity assay by using PDK1, AKT1, Src, and MARK4 as enzyme. Compared with MARK4 (IC_50_: 1.301 μM), compound **50** was insensitive to PDK1, AKT1, and Src with the IC_50_ values of 5.973, 11.762, and 19.018 μM, respectively, while BX-795 showed similar activity against these kinases, suggesting good selectivity of compound **50** (Table 2).

**Table 1:** Kinase activity assay of coumarins.

**Cpd**.	**IC_**50**_ (μM)^**a**^**	**Cpd**.	**IC_**50**_ (μM)^**a**^**	**Cpd**.	**IC_**50**_ (μM)^**a**^**	**Cpd**.	**IC_**50**_ (μM)^**a**^**
**1**	>50	**18**	30.992 ± 2.813	**35**	12.761 ± 1.245	**52**	27.034 ± 3.647
**2**	>50	**19**	27.004 ± 2.384	**36**	33.558 ± 2.749	**53**	24.9734 ± 2.402
**3**	>50	**20**	>50	**37**	37.805 ± 5.430	**54**	21.252 ± 1.995
**4**	40.982 ± 5.401	**21**	25.503 ± 1.007	**38**	40.916 ± 4.036	**55**	>50
**5**	35.016 ± 3.732	**22**	27.981 ± 2.012	**39**	16.883 ± 4.264	**56**	>50
**6**	>50	**23**	17.421 ± 2.686	**40**	38.770 ± 4.833	**57**	>50
**7**	>50	**24**	10.042 ± 3.652	**41**	>50	**58**	>50
**8**	37.872 ± 3.447	**25**	7.763 ± 2.931	**42**	>50	**59**	>50
**9**	>50	**26**	>50	**43**	>50	**60**	>50
**10**	25.127 ± 5.806	**27**	42.073 ± 3.989	**44**	>50	**61**	>50
**11**	29.705 ± 4.211	**28**	>50	**45**	>50	**62**	>50
**12**	22.848 ± 4.605	**29**	37.418 ± 2.840	**46**	>50	**63**	>50
**13**	8.975 ± 2.739	**30**	39.342 ± 3.119	**47**	14.609 ± 2.992	**64**	40.363 ± 2.867
**14**	34.467 ± 3.298	**31**	35.009 ± 5.782	**48**	2.097 ± 0.372	**65**	36.112 ± 1.652
**15**	31.502 ± 2.285	**32**	37.722 ± 6.071	**49**	4.121 ± 0.605	**66**	35.603 ± 3.902
**16**	>50	**33**	46.340 ± 3.043	**50**	1.301 ± 0.102	**67**	11.086 ± 0.786
**17**	>50	**34**	>50	**51**	25.854 ± 2.732	**68**	32.182 ± 2.753

a *The experiments were run in duplicate and shown as means ± SEM*.

**Table 2:** Kinase assay of molecules using PDK1, AKT1, Src, or MARK4 as enzyme.

**Kinase**	**IC**_****50****_ **value in kinase activity assay (μM)**^****a****^	
	**Cpd. 50**	**BX-795**
PDK1	5.973 ± 0.289	1.684 ± 0.192
AKT1	11.762 ± 0.336	2.866 ± 0.269
Src	19.018 ± 1.323	5.871 ± 0.405
MARK4	1.301 ± 0.102	4.994 ± 0.812

a *The experiments were run in duplicate and shown as means ± SEM*.

Based on the data of cell viability assay (Figure 2A and Table 1), we subsequently analyzed the structure-activity relationships (SARs) of coumarins as MARK4 inhibitor. The SARs could be summarized as follow: (1) the substitutions at the C-4 position of A ring were unfavorable: **55–64**; (2) the aryl or ketone at C-3 position of A ring were favorable: **13**, **24**, **25**, **35**, **47**–**50**; (3) B ring of coumarin should be substituted: **48–50** vs. **51–53**; (4) Sulfur or oxygen at X-1 position of A ring was similar: **18** vs. **67**, **19** vs. **66**, **21** vs. **65** and **22** vs. **68**. This conclusion would give helpful clues for structural optimization of coumarin as MARK4 inhibitors.

To further check the *in vitro* interaction between MARK4 and compound **50**, we performed surface plasmon resonance (SPR) assay and thermal shift assay to characterize the protein-small molecule interaction. As in Figure 2D, compound **50** showed strong binding affinity toward MARK4 with a dissociation constant (K_d_ value) of 0.346 μM in the SPR assay with different sample concentrations. This affinity was greater than that between BX-795 and MARK4 (K_d_: 6.46 μM) (Naz et al., 2015), interpreting the improved activity of compound **50** over BX-795. Furthermore, compound **50** significantly increased the thermal stability of MARK4 with a temperature shift of 3.13°C, indicating the direct binding of compound **50** and MARK4 *in vitro* (Figure 2E).

**Figure 3:**
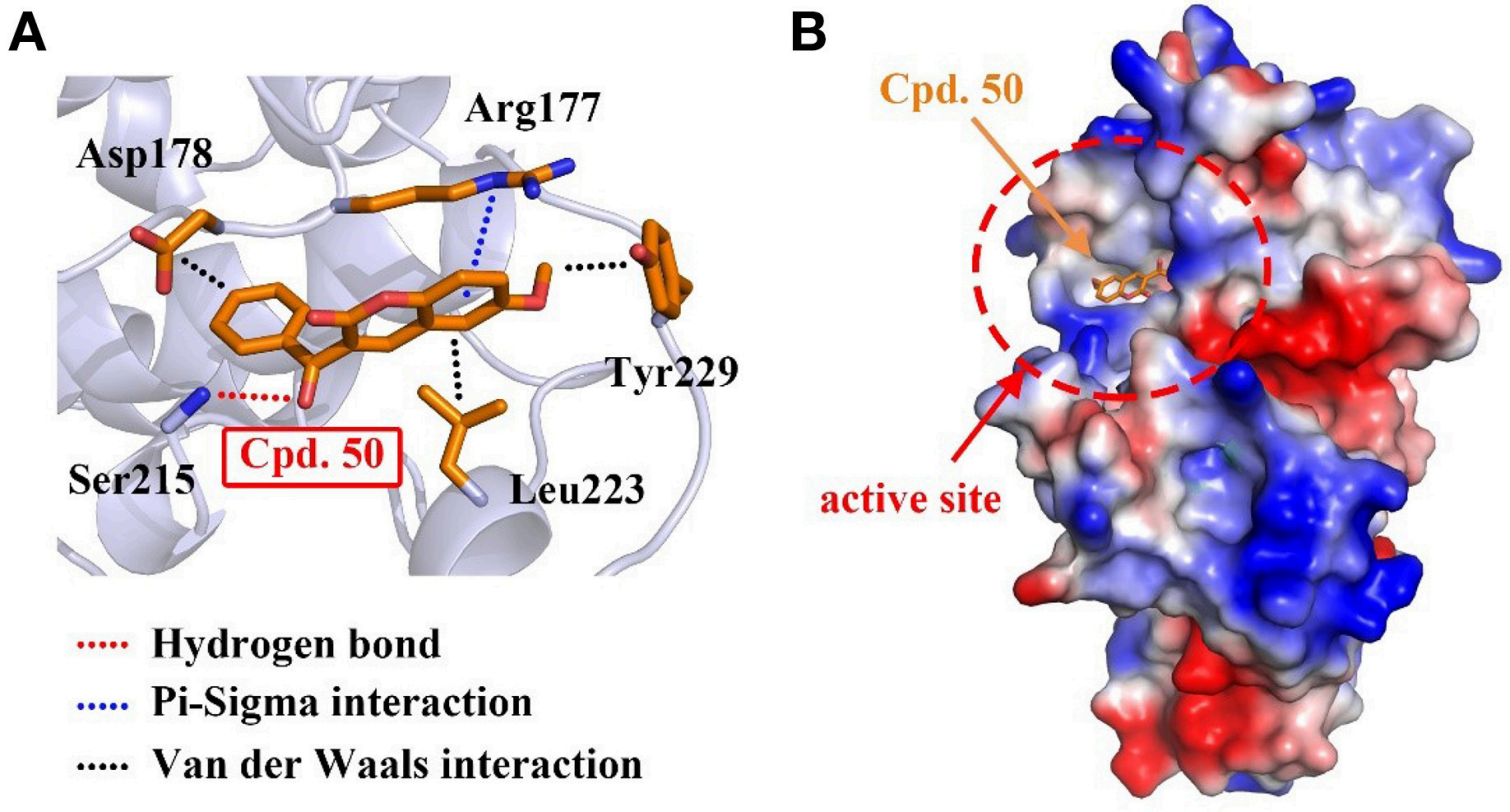
Compound **50** located in the active site of MARK4. **(A)** Flexible docking of compound **50** in complex with MARK4 (PDB ID: 5ES1). **(B)** Flexible docking results of MARK4 (PDB ID: 5ES1) and compound **50**. The image represented the charge distribution of MARK4 in complex with compound **50**.

To interpret the structural basis of MARK4-compound **50** interaction, we simulated the binding pattern of MARK4 and compound **50** via the flexible docking in Discovery Studio v3.1 software. The data showed compound **50** directly located into the active site of MARK4 through the amino acid residues of Arg177 and Ser215 by hydrogen bond and π-sigma interaction, respectively (Figures 3A,B). Additionally, Van der Waals interactions between compound **50** and residues including Asp178, Leu223, and Tyr229, also enhanced the association of MARK4 and compound **50** (Figure 3A). These data provide the experimental and *in silico* evidence for the MARK4-compound **50** interaction.

**Figure 4:**
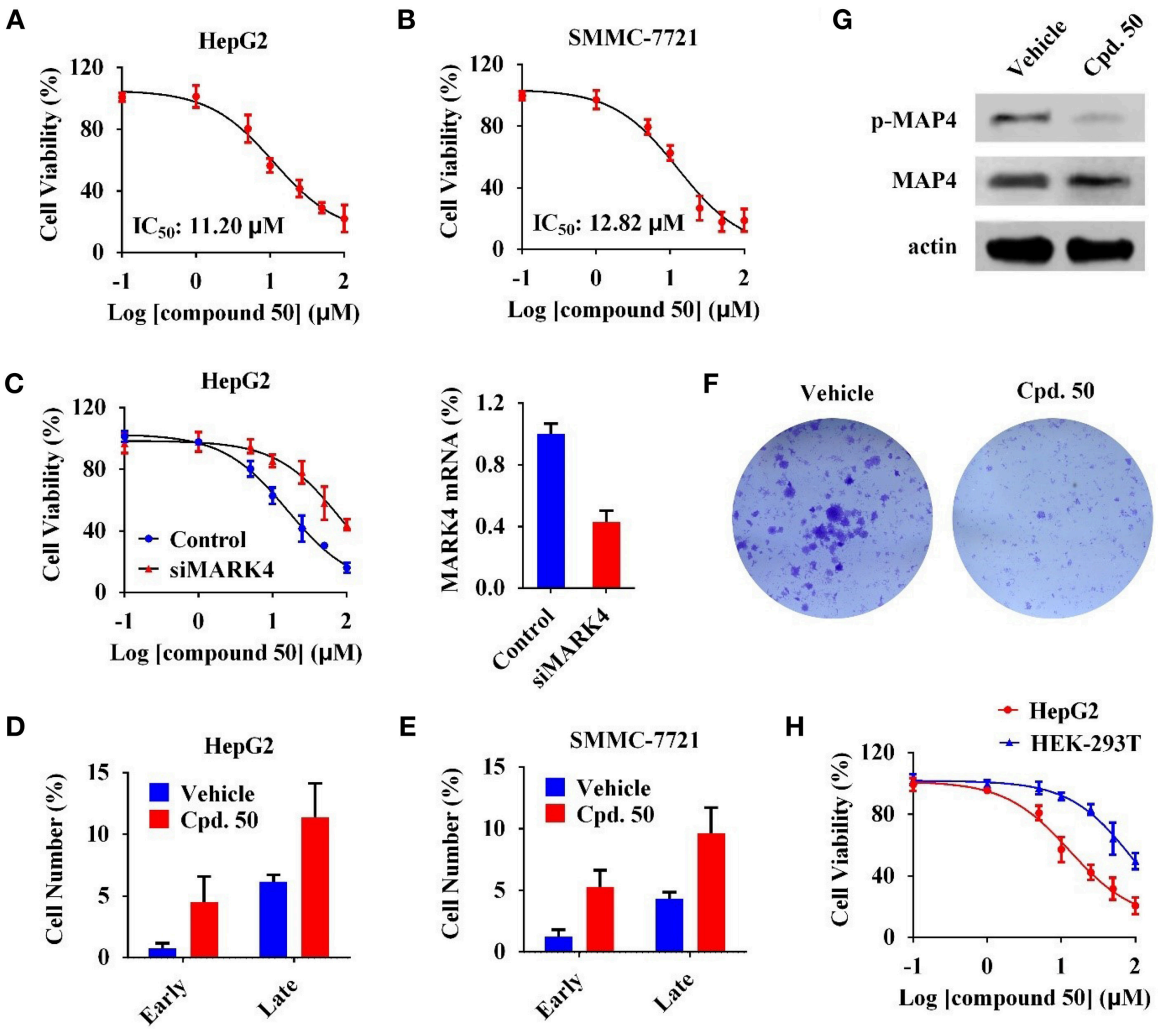
Compound **50** suppressed cell viability and induced apoptosis of HCC cells in a MARK4-dependent manner. **(A,B)** Cell viability assay of compound **50** in **(A)** HepG2 and **(B)** SMMC-7721 cells. **(C)** (left) Cell viability assay of compound **50** in HepG2 cells transiently transfected MARK4-knockdown and control vectors. (right) MARK4-knowdown was confirmed by real-time quantitative PCR. **(D,E)** Cell apoptosis assay of compound **50** (15 μM) in **(D)** HepG2 and **(E)** SMMC-7721 cells. **(F)** Colony formation assay of HepG2 cells with or without compound **50** treatment. **(G)** Immunoblotting against p-MAP4, MAP4, and actin antibodies of HepG2 cell lysates with or without the treatment of compound **50** (15 μM). **(H)** Cell viability assay of compound **50** in HepG2 and HEK-293T cells.

We subsequently characterized function of compound **50** in HCC cells. In cell viability assay, compound **50** suppressed cell viability in HepG2 and SMMC-7721 cells with the IC_50_ values of 11.20 and 12.82 μM, respectively (Figures 4A,B). MARK4-knockdown (Figure 4C, right) significantly attenuated the anti-proliferative activity of compound **50** in HepG2 cells (Figure 4C, left), suggesting a favorable MARK4 specificity of compound **50** in HCC cells. It is reported that MARK4 participates the regulation of apoptosis (Heidary Arash et al., 2017). Thus, we investigated the influence of compound **50** on apoptosis via flow cytometry. The results indicated compound **50**, in accordance with previous work, obviously increased the cell numbers of both early and late apoptotic cells, inducing apoptosis in both HepG2 and SMMC-7721 cells (Figures 4D,E). In colony formation assay, compared with the treatment of vehicle, compound **50** significantly suppressed the colony formation of HepG2 cells, further supporting the HCC-preventing function of compound **50** (Figure 4F). Moreover, to evaluate the downstream of MARK4 inhibition by compound **50**, we performed immunoblotting analysis in the lysates of HepG2 cells with or without compound **50** incubation. As in Figure 4G, compound **50** decreased the level of MAP4 (the substrate of MARK4 in HCC) (Sun et al., 2016) phosphorylation, suggesting the downstream of MARK4 inhibition in HCC cells. In addition, we performed the cell viability assay in human normal HEK-293T cells to characterize the toxicity of compound **50**. Because the cell viability of HEK-293T cells was suppressed only at the high concentrations (>50 μM), the biological function of compound **50** was significantly attenuated in HEK-293T cells than HCC cells (Figure 4H), indicating a limited toxicity of compound **50** in normal cells. These data suggested compound **50** suppress cell viability and induce apoptosis of HCC cells in a MARK4-dependent manner.

**Figure 5:**
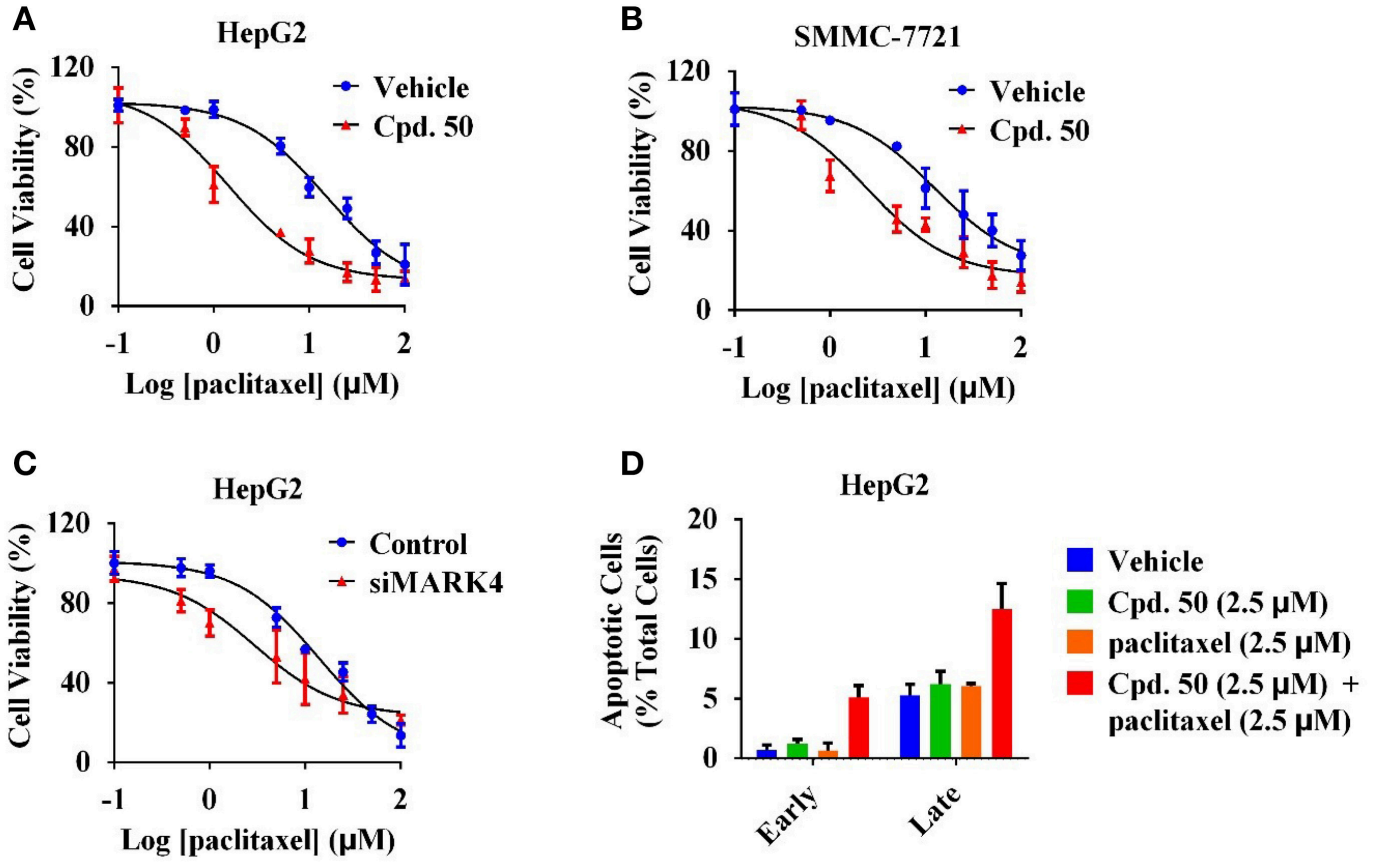
Compound **50** sensitized HCC cells to paclitaxel treatment. **(A)** Cell viability assay of the varied concentrations of paclitaxel in HepG2 cells with or without the incubation of compound **50** (2.5 μM). **(B)** Cell viability assay of the varied concentrations of paclitaxel in SMMC-7721 cells with or without the incubation of compound **50** (2.5 μM). **(C)** Cell viability assay of the varied concentrations of paclitaxel in HepG2 cells with the transiently transfection of MARK4-knockdown or control vectors. **(D)** Cell apoptosis assay of paclitaxel (2.5 μM) in HepG2 cells with or without the incubation of compound **50** (2.5 μM).

Our previous work has revealed that MARK4 phosphorylated MAP4 to increase microtubule dynamics and confer paclitaxel resistance in HCC (Sun et al., 2016), giving MARK4 as an attractive target to overcome paclitaxel resistance. To answer whether MARK4 inhibition was able to sensitize HCC cells to paclitaxel, we incubated HepG2 and SMMC-7721 cells with 2.5 μM of compound **50**. The cell viability of HCC cells upon 2.5 μM of compound **50** treatment was more than 80% (Figures 4A,B), indicating compound **50** was inactive against HCC cells at this concentration solely. Intriguingly, the inhibitory activity of paclitaxel in HCC was remarkably enhanced in the presence of compound **50** (Figures 5A,B). In accord with MARK4 inhibition by compound **50**, paclitaxel also had an increased sensitivity in MARK4-knockdown HepG2 cells (Figure 5C), suggesting the therapeutic role of MARK4 in paclitaxel-resisted HCC. Because paclitaxel has an ability to induce apoptosis of human solid tumors, we analyzed the effect of paclitaxel in apoptosis of HCC cells upon compound **50** exposure. As in Figure 5D, both paclitaxel and compound **50** were unable to trigger distinct apoptosis at the concentration of 2.5 μM. Paclitaxel (2.5 μM) in combination with compound **50** (2.5 μM) activated HCC apoptosis (Figure 5D), providing further evidence for the conclusion that compound **50** sensitized HCC cells to paclitaxel treatment.

Many investigations suggested the oncogenic role of MARK4 in human cancer, arousing a great interest to develop small-molecule inhibitors for targeted therapies. In the past few years, a series of MARK4 inhibitors, such as 9-oxo-9H-acridin-10-yl, pyrazolopyrimidine, pyrrolopyrimidinone, isatin-triazole hydrazone and 2-heteroarylchromone, were discovered, suggesting new molecular tools and/or drug candidates for cancer research and therapy (Jenardhanan et al., 2014; Sack et al., 2016; Katz et al., 2017; Khan et al., 2017; Mohammad et al., 2018; Parveen et al., 2018; Aneja et al., 2019). But the limited potency and specificity of these known MARK4 inhibitors make these molecules unfavorable solely as clinical drug for the treatment of cancers, including HCC. Paclitaxel, with clinical safety and efficacy, is an FDA-approved drug against multiple solid tumors, but paclitaxel is commonly resisted in HCC patients. Our recent work indicates that, due to the downregulation of miR-122 in HCC, the MARK4-mediated MAP4 detachment is activated, resulting in the augment of microtubule dynamics and paclitaxel resistance (Sun et al., 2016). Thus, MARK4 inhibition has the potential to attenuate paclitaxel resistance. In this work, we demonstrated that MARK4 inhibitor has the ability to enhance the sensitivity of paclitaxel toward HCC cells (Figure 5), providing a novel insight for HCC treatment and enlarging the potential application for paclitaxel. Except for MARK4 inhibition, the treatment increasing the expression of miR-122, such as Pin1-targeted intervention (Li et al., 2018; Pu et al., 2018; Zheng et al., 2019), is also a theoretically feasible proposal to sensitize paclitaxel in HCC, which should be considered in further study.

In the past few years, the coumarin analogs, both natural and synthetic origins, were identified as lead compounds exhibiting attractive anticancer activity (Emani and Dadashpour, 2015; Venkata Sairam et al., 2016). For instance, gemcitabine-coumarin-biotin conjugates were developed as a target specific theranostic anticancer prodrug, wherein both a therapeutic effect and drug uptake can be readily monitored by two photon fluorescence imaging (Maiti et al., 2013). 4-Substituted coumarin derivative SKLB060 was discovered as novel tubulin inhibitor, inhibiting tubulin polymerization and subsequently inducing G2/M cell cycle arrest and apoptosis in cancer cells (Yan et al., 2018). In this work, we revealed that coumarin-based compound **50** targted MARK4 kinase to sentisize HCC cells to paclitaxel treatment, enlarging the therapeutical potential of coumarins in human cancers. Furthermore, diverse synthetic methodologies have been established to prepare coumarin scaffold (Pereira et al., 2018), facilitating the structural optimization and drug development of coumarins.

## Conclusion

In summary, the preliminary results of computer-aided drug design enlightened us that coumarin derivatives could be potential small-molecule MARK4 inhibitors. Thus, we prepared a series of coumarin derivatives via multiple synthetic methodologies and discovered that compound **50** with coumarin skeleton was a novel MARK4 inhibitor via both computer-aided virtual screening and intensive biological evaluation. This compound suppressed HCC cell viability and induced apoptosis in a MARK4-dependent manner. Importantly, compound **50** significantly increased the drug response of paclitaxel treatment to HCC cells, which were ordinarily resisted to paclitaxel. Our discovery offers a promise solution for HCC therapy by use of paclitaxel effectively.

## Materials and Methods

### Computer-Aided Virtual Screening and Flexible Docking

In Discovery Studio v3.1 software, we established a virtual screening model based on the crystal structure of MARK4 catalytic domain (PDB ID: 5ES1), and performed molecular docking and flexible docking following GOLD docking and Flexible docking protocols, respectively. The images of molecular docking results were processed by PyMoL v1.8 software.

### Chemical Synthesis

#### Synthesis of 1–46

A mixture of salicylaldehyde derivative (8 mmol), acetic anhydride (20 mL) and triethylamine (5 mL) were heated at 120°C for 8 h. After cooling to room temperature, the reaction mixture was slowly poured into ice-water (200 mL) with violent stirring. The precipitated solid was filtered off and recrystallized from ethyl acetate to give coumarin **1–10**. The characterizations of coumarin **1**–**10** were previous reported in references (Scheme 1) (Takaishi et al., 2008; Wei et al., 2013; Meng et al., 2017). The preparation and characterizations of 3-arylcoumarins **11–46** was described in our previous work (Pu et al., 2014a,b).

#### Synthesis of 47–54

The preparation of **47–54** was under the procedures described in previous report (Karade et al., 2008). In brief, a mixture of 2-hydroxybenzaldehyde (3 mmol), reactive methylene compound (3 mmol), and L-proline (10 mol%) was heated under neat conditions for 0.5 h. The reaction was monitored by TLC. After completion of reaction, the reaction mixture was cooled and recrystallized from ethanol to obtain 3-substituted coumarin derivatives **47–54** with excellent yields. The characterizations of coumarin **47**–**54** were previous reported in references (Scheme 2) (Dean and Park, 1976; Sugino and Tanaka, 2001; Rao and Sivakumar, 2006; Karade et al., 2008).

#### Synthesis of 55–64

The synthesis of **55–57** was under the procedures described in previous report (Smitha and Sanjeeva Reddy, 2004). In brief, a mixture of phenolic substrate (10 mmol) and keto ester (10 mmol) was heated at 70°C in the presence of zirconium (IV) chloride (46 mg, 2 mol%). After completion of the reaction, the reaction mixture was cooled to room temperature and poured into crushed ice. The solid was filtered off, washed with ice-cold water, and recrystallized from ethanol to obtain the pure product **55–57** (Scheme 3).

To a mixture of **56** (**57** or **62**, 3 mmol) (Serra et al., 2012) and potassium carbonate (30 mmol) in DMF (10 mL) was added iodomethane (9 mmol). The mixture was stirred at ambient temperature overnight. The mixture was poured into water (100 mL) and extracted with ethyl acetate. The combined organic layer was washed successively with water and brine, dried over anhydrous sodium sulfate, and concentrated under reduced pressure. The desired product **58** (**60** or **64**) was isolated using silica gel column chromatography (Schemes 3, 4).

To a mixture of **56** (**57** or **62**, 3 mmol) and potassium carbonate (30 mmol) in DMF (10 mL) was added benzyl chloride (9 mmol). The mixture was stirred at 60°C until the reaction was completed. The mixture was poured into water (100 mL) and extracted with ethyl acetate. The combined organic layer was washed successively with water and brine, dried over anhydrous sodium sulfate, and concentrated under reduced pressure. The desired product **59** (**61** or **63**) was isolated using silica gel column chromatography (Schemes 3, 4). The characterizations of coumarin **55**–**64** were previous reported in references (Smitha and Sanjeeva Reddy, 2004; Takaishi et al., 2008; Cavar et al., 2009; Sun et al., 2011; Zhang et al., 2011; Serra et al., 2012; Olmedo et al., 2017).

#### Synthesis of 65–68

The synthesis of **65–68** was under the procedures described in previous report (Meth-Cohn and Tarnowski, 1978). In brief, a mixture of 2-*t*-butylthiobenzaldehydes (10 mmol), phenyl acetonitrile derivatives (1.6 g), sodium ethoxide solution in ethanol (0.6 mL, 20% w/v) and ethanol (5 mL) is heated on a boiling water bath for 10 min, then cooled and diluted with water, filtered to give the styrene intermediates (**p65–p68**). The styrene in polyphosphoric acid (40 g) is heated at 100°C for 0.8 h. The mixture is cooled and diluted with water, extracted with ethyl ether. The water phase was diluted with water and heated for 4 h. The mixture was cooled and filtered. The crude products were recrystallized from ethanol to give **65–68** (Scheme 5). The characterization of coumarin **65** was previous reported in references (Nelson, 2004). The copies of NMR spectra of coumarin 66–68 and their precursor P66-P68, see Supplementary Material.

(Z)-3-(2-(Tert-butylthio)phenyl)-2-(4-methoxyphenyl)acrylonitrile (**p66**): ^1^H NMR (400 MHz, CDCl_3_) δ 8.26 (s, 1H), 8.13 (dd, *J* = 7.8, 1.4 Hz, 1H), 7.69–7.62 (m, 3H), 7.50 (td, *J* = 7.6, 1.4 Hz, 1H), 7.40 (td, *J* = 7.5, 1.4 Hz, 1H), 7.02–6.96 (m, 2H), 3.87 (s, 3H), 1.27 (s, 9H). ^13^C NMR (100 MHz, CDCl_3_) δ 160.47, 140.82, 139.91, 139.34, 133.65, 129.75, 129.61, 128.84, 127.42, 126.85, 117.96, 114.49, 112.53, 55.48, 48.31, 31.13. HR-ESIMS: 346.1239 [M+Na]^+^ (calc. for C_20_H_21_NNaOS, 346.1237).

(Z)-3-(2-(Tert-butylthio)phenyl)-2-(3,4-dimethoxyphenyl)acrylonitrile (**p67**): ^1^H NMR (400 MHz, CDCl_3_) δ 8.28 (s, 1H), 8.17–8.11 (m, 1H), 7.66 (dd, *J* = 7.7, 1.4 Hz, 1H), 7.51 (td, *J* = 7.7, 1.4 Hz, 1H), 7.41 (td, *J* = 7.5, 1.5 Hz, 1H), 7.31 (dd, J = 8.4, 2.2 Hz, 1H), 7.20 (d, *J* = 2.2 Hz, 1H), 6.95 (d, *J* = 8.4 Hz, 1H), 3.97 (s, 3H), 3.94 (s, 3H), 1.28 (s, 9H). ^13^C NMR (100 MHz, CDCl_3_) δ 150.08, 149.28, 141.14, 139.80, 139.36, 133.68, 129.83, 129.66, 128.80, 127.22, 119.09, 117.93, 112.60, 111.32, 108.81, 56.07, 56.01, 48.31, 31.14. HR-ESIMS: 376.1349 [M+Na]^+^ (calc. for C_21_H_23_NNaO_2_S, 376.1342).

(Z)-3-(2-(Tert-butylthio)phenyl)-2-(4-fluorophenyl)acrylonitrile (**p68**): ^1^H NMR (400 MHz, CDCl_3_) δ 8.31 (s, 1H), 8.17–8.11 (m, 1H), 7.73–7.64 (m, 3H), 7.52 (td, *J* = 7.6, 1.4 Hz, 1H), 7.43 (td, *J* = 7.5, 1.5 Hz, 1H), 7.20–7.13 (m, 2H), 1.28 (s, 9H). ^13^C NMR (100 MHz, CDCl_3_) δ163.25 (d, *J* = 250.2 Hz), 142.84, 142.82, 139.49, 139.37, 133.88, 130.46 (d, *J* = 3.4 Hz), 130.16, 129.68, 128.86, 127.92 (d, *J* = 8.4 Hz), 117.65, 116.23 (d, *J* = 22.0 Hz), 111.92, 48.40, 31.12. HR-ESIMS: 334.1036 [M+Na]^+^ (calc. for C_19_H_18_FNNaS, 334.1037).

3-(4-Methoxyphenyl)-2H-thiochromen-2-one **(66)**: Yield: 79%. ^1^H NMR (400 MHz, CDCl_3_) δ 7.76 (s, 1H), 7.64 (d, *J* = 7.7 Hz, 1H), 7.52 (d, *J* = 8.7 Hz, 2H), 7.49–7.44 (m, 2H), 7.43–7.35 (m, 1H), 6.97 (d, *J* = 8.8 Hz, 2H), 3.85 (s, 3H). ^13^C NMR (100 MHz, CDCl_3_) δ 185.17, 159.89, 141.94, 137.46, 135.18, 131.55, 130.38, 129.28, 127.91, 127.03, 126.42, 125.12, 113.82, 55.39. HR-ESIMS: 291.0450 [M+Na]^+^ (calc. for C_16_H_12_NaO_2_S, 291.0451).

3-(3,4-Dimethoxyphenyl)-2H-thiochromen-2-one **(67)**: Yield: 71%. ^1^H NMR (400 MHz, CDCl_3_) δ 7.78 (s, 1H), 7.65 (d, *J* = 7.8 Hz, 1H), 7.51–7.44 (m, 2H), 7.39 (ddd, *J* = 8.3, 5.9, 2.6 Hz, 1H), 7.12 (d, *J* = 7.1 Hz, 2H), 6.93 (d, *J* = 8.6 Hz, 1H), 3.92 (d, *J* = 1.4 Hz, 6H). ^13^C NMR (100 MHz, CDCl_3_) δ 185.16, 149.43, 148.54, 142.16, 137.46, 135.24, 131.61, 129.38, 128.26, 126.96, 126.46, 125.12, 121.70, 112.40, 110.96, 56.02, 55.98. HR-ESIMS: 321.0556 [M+Na]^+^ (calc. for C_17_H_14_NaO_3_S, 321.0556).

3-(4-Fluorophenyl)-2H-thiochromen-2-one **(68)**: Yield: 65%. ^1^H NMR (400 MHz, CDCl_3_) δ 7.78 (s, 1H), 7.69–7.63 (m, 1H), 7.59–7.45 (m, 4H), 7.45–7.37 (m, 1H), 7.16–7.09 (m, 2H). ^13^C NMR (100 MHz, CDCl_3_) δ 184.88, 162.89 (d, *J* = 248.3 Hz), 142.72, 137.69, 134.60, 131.75, 131.56 (d, *J* = 3.4 Hz), 130.94 (d, *J* = 8.2 Hz), 129.67, 126.74, 126.56, 125.23, 115.37 (d, *J* = 21.6 Hz). HR-ESIMS: 279.0255 [M+Na]^+^ (calc. for C_15_H_9_FNaOS, 279.0251).

### Expression and Purification of MARK4

MARK4 catalytic domain sequence was subcloned into pET-28a vector. Protein expression in *E. coli*. BL21 (DE3) was induced by IPTG overnight at 16°C, and the cells were then pelleted by centrifugation at 4,000 rpm for 15 min and resuspended in 20 mL buffer (20 mM Tris-HCl, 150 mM NaCl, 25 mM imidazole pH 8.0). Proteins were purified by nickel-affinity chromatography followed by thrombin cleavage. The proteins were further purified by size-exclusion chromatography using a Superdex 75 column (GE Healthcare).

### Kinase Activity Assay

The kinase activity of MARK4 was measured via ADP-Glo™ kinase assay kit (Promega, USA) under manufacturer's instruction. The recombinant MARK4 catalytic domain was used as kinase. MARK4 (5 nM) activity was tested in a reaction (10 μL) containing 25 mM Tris (pH 7.6), 10 mM MgCl_2_, 150 mM NaCl, 1 mM EDTA, 1 mM DTT, 100 μM ATP, 5 μM MAP4 and varied concentrations of samples for 30 min. The reaction progress was monitored on Thermo Scientific Varioskan Flash Multimode Reader. Each data point was collected in duplicate and kinetic parameters were obtained using GraphPad Prism v6.0 software.

### Surface Plasmon Resonance (SPR)

The interaction of small molecule with MARK4 was monitored by surface plasmon resonance (SPR) using a BIAcore T100 instrument (GE Healthcare, USA). In brief, recombinant MARK4 catalytic domain was immobilized on a carboxyl methylated dextran sensor chip (Sensor Chip CM_3_). A flow channel blocked by ethanolamine was used as control surface. The specific interaction of samples with the immobilized MARK4 catalytic domain was assessed. All samples were analyzed at a flow rate of 30 μL/min with 10 mM HEPES running buffer and contact time of 260 s. The surface was washed and regenerated with a 10 mM glycine-HCl buffer at pH 3.0 followed by a 30 min waiting time for dissolution after each experiment. The analyses were performed in BIAcore T100 evaluation software, version 2.0.2 (GE Healthcare, USA).

### Thermal Shift Assay

Recombinant MARK4 catalytic domain was diluted with PBS and divided into two aliquots, with one aliquot being treated with sample (1 μM) and the other aliquot with vehicle. After 20 min of incubation at room temperature, the respective mixtures were divided into smaller (40 μL) aliquots and heated individually at different temperatures for 3 min, followed by cooling at room temperature for 3 min. The appropriate temperatures were determined in preliminary CETSA experiments. The heated mixtures were centrifuged at 20,000 × g at 4°C for 20 min in order to separate the soluble fractions from precipitates. The supernatants were transferred to new microtubes and analyzed by HPLC.

### Cell Culture and Transfection

HepG2 and SMMC-7721 cells were cultured in DMEM medium (Gibco) supplemented with 10% FBS (Gibco, USA). For transient transfection, cells were transfected with DNA by Lipofectamine 2000 (Thermo fisher, USA).

### Cell Viability Assay

HCC cells (100 μL/each well, 20,000 cells/mL) were seeded in a 96-well culture plate and allowed to grow for 24 h in DMEM medium supplemented with 10% FBS and 1% penicillin/streptomycin sulfate. The cells were then treated with drugs or vehicle control and incubated in a 5% CO_2_ incubator for indicated time. At the end of incubation, 10 μL of MTT stock solution (5 mg/mL) was added into each well. The plate was continued to be incubated at 37°C for 4 h before the medium was removed. Then, DMSO (100 μL) was added into each well, followed by thorough shaking. The absorbance of the formazan product was measured at 570 nm on Thermo Scientific Varioskan Flash Multimode Reader. The IC_50_ value was obtained by fitting dose-response data to a three parametric non-linear regression model using GraphPad Prism 6.0 software.

### Cell Apoptosis Assay

HCC cells (2 × 10^5^ cells/well) were plated in a 6-well plate and treated with samples at 37°C for 36 h. After incubation, the cells were harvested and washed with ice-cold PBS. Annexin *V-FITC-PI* apoptosis detection kit (Abcam, USA) was used for a direct observation of viable, early apoptotic, and late apoptotic cells. Annexin V-FITC and propidium iodide were added according to the manufacturer's instructions, and the samples were incubated in the dark for 15 min. The apoptosis ratio was analyzed using BD FACSCalibur flow cytometry.

### Immunoblotting

HepG2 cells (2 × 10^5^ cells/well) were plated in a 6-well plate and treated with compound **50** (15 μM) and vehicle at 37°C for 36 h. Cells were harvested, washed with physiological saline, and lysed with RIPA lysis buffer (Beyotime) including phosphatase and protease cocktail (Bimake). Protein was quantified by BCA protein assay kit (Bestbio) and subjected to SDS-PAGE. The separated proteins were transferred onto PVDF membranes and probed with antibody (anti-p-MAP4, anti-MAP4, and anti-actin) and peroxidase-conjugated secondary antibody, which was followed by enhanced chemiluminescence detection (Amersham Biosciences).

## Data Availability

The raw data supporting the conclusions of this manuscript will be made available by the authors, without undue reservation, to any qualified researcher.

## Author Contributions

All authors listed have made a substantial, direct and intellectual contribution to the work, and approved it for publication.

### Conflict of Interest Statement

The authors declare that the research was conducted in the absence of any commercial or financial relationships that could be construed as a potential conflict of interest.

## Supplementary Material

The Supplementary Material for this article can be found online at: https://www.frontiersin.org/articles/10.3389/fchem.2019.00366/full#supplementary-material

## References

[B1] Aneja B. Khan N. S. Khan P. Queen A. Hussain A. Rehman M. T.. (2019). Design and development of Isatin-triazole hydrazones as potential inhibitors of microtubule affinity-regulating kinase 4 for the therapeutic management of cell proliferation and metastasis. Eur. J. Med. Chem. 163, 840–852. 10.1016/j.ejmech.2018.12.026 30579124

[B2] Bandiera S. Pfeffer S. Baumert T. F. Zeisel M. B. (2015). miR-122–a key factor and therapeutic target in liver disease. J. Hepatol. 62, 448–457. 10.1016/j.jhep.2014.10.004 25308172

[B3] Beghini A. Magnani I. Roversi G. Piepoli T. Di Terlizzi S. Moroni R. F.. (2003). The neural progenitor-restricted isoform of the MARK4 gene in 19q13.2 is upregulated in human gliomas and overexpressed in a subset of glioblastoma cell lines. Oncogene 22, 2581–2591. 10.1038/sj.onc.1206336 12735302

[B4] Bharadwaj R. Yu H. (2004). The spindle checkpoint, aneuploidy, and cancer. Oncogene 23, 2016–2027. 10.1038/sj.onc.1207374 15021889

[B5] Bray F. Ferlay J. Soerjomataram I. Siegel R. L. Torre L. A. Jemal A. (2018). Global cancer statistics 2018: globocan estimates of incidence and mortality worldwide for 36 cancers in 185 countries. CA Cancer J. Clin. 68, 394–424. 10.3322/caac.21492 30207593

[B6] Brito D. A. Yang Z. Rieder C. L. (2008). Microtubules do not promote mitotic slippage when the spindle assembly checkpoint cannot be satisfied. J. Cell Biol. 182, 623–629. 10.1083/jcb.200805072 18710927 PMC2518701

[B7] Cavar S. Kovač F. Maksimović M. (2009). Synthesis and antioxidant activity of selected 4-methylcoumarins. Food Chem. 117, 135–142. 10.1016/j.foodchem.2009.03.087

[B8] Chao Y. Chan W. K. Birkhofer M. J. Hu O. Y. Wang S. S. Huang Y. S.. (1998). Phase II and pharmacokinetic study of paclitaxel therapy for unresectable hepatocellular carcinoma patients. Br. J. Cancer 78, 34–39. 10.1038/bjc.1998.438 9662247 PMC2062942

[B9] Dean F. M. Park K. B. (1976). Activating groups for the ring expansion of coumarin by diazoethane: benzoyl, pivaloyl, arylsulphonyl, arylsulphinyl, and nitro. J. Chem. Soc. Perkin 1, 1260–1268. 10.1039/P19760001260

[B10] Drewes G. Ebneth A. Preuss U. Mandelkow E. M. Mandelkow E. (1997). MARK, a novel family of protein kinases that phosphorylate microtubule-associated proteins and trigger microtubule disruption. Cell 89, 297–308. 10.1016/S0092-8674(00)80208-1 9108484

[B11] Emani S. Dadashpour S. (2015). Current developments of coumarin-based anti-cancer agents in medicinal chemistry. Eur. J. Med. Chem. 102, 611–630. 10.1016/j.ejmech.2015.08.033 26318068

[B12] Feldman R. I. Wu J. M. Polokoff M. A. Kochanny M. J. Dinter H. Zhu D.. (2005). Novel small molecule inhibitors of 3-phosphoinositide-dependent kinase-1. J. Biol. Chem. 280, 19867–19874. 10.1074/jbc.M501367200 15772071

[B13] Galle P. R. (2008). Sorafenib in advanced hepatocellular carcinoma-We have won a battle not the war. J. Hepatol. 49, 871–873. 10.1016/j.jhep.2008.09.001 18817997

[B14] Goodwin J. M. Svensson R. U. Lou H. J. Winslow M. M. Turk B. E. Shaw R. J. (2014). An AMPK-independent signaling pathway downstream of the LKB1 tumor suppressor controls Snail1 and metastatic potential. Mol. Cell 55, 436–450. 10.1016/j.molcel.2014.06.021 25042806 PMC4151130

[B15] Heidary Arash E. Shiban A. Song S. Attisano L. (2017). MARK4 inhibits Hippo signaling to promote proliferation and migration of breast cancer cells. EMBO Rep. 18, 420–436. 10.15252/embr.201642455 28183853 PMC5331264

[B16] Jenardhanan P. Mannu J. Mathur P. P. (2014). The structural analysis of MARK4 and the exploration of specific inhibitors for the MARK family: a computational approach to obstruct the role of MARK4 in prostate cancer progression. Mol. Biosyst. 10, 1845–1868. 10.1039/c3mb70591a 24763618

[B17] Jordan M. A. Wilson L. (2004). Microtubules as a target for anticancer drugs. Nat. Rev. Cancer 4, 253–265. 10.1038/nrc1317 15057285

[B18] Karade N. N. Gampawar S. V. Shinde S. V. Jadhav W. N. (2008). L-proline catalyzed solvent-free knoevenagel condensation for the synthesis of 3-substituted coumarins. Chin. J. Chem. 39, 1686–1689. 10.1002/cjoc.200790311

[B19] Kato T. Satoh S. Okabe H. Kitahara O. Ono K. Kihara C.. (2001). Isolation of a novel human gene, MARKL1, homologous to MARK3 and its involvement in hepatocellular carcinogenesis. Neoplasia 3, 4–9. 10.1038/sj/neo/7900132 11326310 PMC1505019

[B20] Katz J. D. Haidle A. Childers K. K. Zabierek A. A. Jewell J. P. Hou Y.. (2017). Structure guided design of a series of selective pyrrolopyrimidinone MARK inhibitors. Bioorg. Med. Chem. Lett. 27, 114–120. 10.1016/j.bmcl.2016.08.068 27816515

[B21] Kavallaris M. (2010). Microtubules and resistance to tubulin-binding agents. Nat. Rev. Cancer 10, 194–204. 10.1038/nrc2803 20147901

[B22] Khan P. Rahman S. Queen A. Manzoor S. Naz F. Hasan G. M.. (2017). Elucidation of dietary polyphenolics as potential inhibitor of microtubule affinity regulating kinase 4: *in silico* and *in vitro* Studies. Sci. Rep. 7:9470. 10.1038/s41598-017-09941-4 28842631 PMC5573368

[B23] Li J. Pu W. Sun H. L. Zhou J. K. Fan X. Zheng Y. (2018). Pin1 impairs microRNA biogenesis by mediating conformation change of XPO5 in hepatocellular carcinoma. Cell Death Differ. 25, 1612–1624. 10.1038/s41418-018-0065-z PMC6143530 29445125

[B24] Llovet J. M. Ricci S. Mazzaferro V. Hilgard P. Gane E. Blanc J. F.. (2008). Sorafenib in advanced hepatocellular carcinoma. N. Engl. J. Med. 359, 378–90. 10.1056/NEJMoa0708857 18650514

[B25] Maiti S. Park N. Han J. H. Jeon H. M. Lee J. H. Bhuniya S.. (2013). Gemcitabine-coumarin-biotin conjugates: a target specific theranostic anticancer prodrug. J. Am. Chem. Soc. 135, 4567–4572. 10.1021/ja401350x 23461361

[B26] Matenia D. Mandelkow E. M. (2009). The tau of MARK: a polarized view of the cytoskeleton. Trends Biochem. Sci. 34, 332–342. 10.1016/j.tibs.2009.03.008 19559622

[B27] Meng J. J. Jiang Q. Li X. Y. Wan L. Fang Z. Guo K. (2017). N-Heterocyclic-carbene-catalyzed redox lactonization of o-hydroxycinnamaldehydes and o-hydroxycinnamyl alcohols to coumarins. Asian J. Org. Chem. 6, 1724–1727. 10.1002/ajoc.201700458

[B28] Meth-Cohn O. Tarnowski B. (1978). A useful synthon for sulfur heterocycles, I. The synthesis of thiocoumarins. Synthesis 1 56–58 10.1055/s-1978-24675

[B29] Mohammad T. Khan F. I. Lobb K. A. Islam A. Ahmad F. Hassan M. I. (2018). Identification and evaluation of bioactive natural products as potential inhibitors of human microtubule affinity-regulating kinase 4 (MARK4). J. Biomol. Struct. Dyn. 37, 1813–1829. 10.1080/07391102.2018.1468282 29683402

[B30] Naz F. Shahbaaz M. Bisetty K. Islam A. Ahmad F. Hassan M. I. (2015). Designing new kinase inhibitor derivatives as therapeutics against common complex diseases: structural basis of microtubule affinity-regulating kinase 4 (MARK4) inhibition. OMICS 19, 700–711. 10.1089/omi.2015.0111 26565604

[B31] Nelson A. (2004). Benzothiopyranones and benzothiopyranthiones. ChemInform 35. 10.1002/chin.200435255

[B32] Olmedo D. A. López-Pérez J. L. Del Olmo E. Bedoya L. M. Sancho R. Alcamí J.. (2017). Neoflavonoids as inhibitors of HIV-1 replication by targeting the Tat and NF-kappaB pathways. Molecules 22:E321. 10.3390/molecules22020321 28218730 PMC6155902

[B33] Parveen I. Khan P. Ali S. Hassan M. I. Ahmed N. (2018). Synthesis, molecular docking and inhibition studies of novel 3-N-aryl substituted-2-heteroarylchromones targeting microtubule affinity regulating kinase 4 inhibitors. Eur. J. Med. Chem. 159:166–177. 10.1016/j.ejmech.2018.09.030 30290280

[B34] Pereira T. M. Franco D. P. Vitorio F. Kummerle A. E. (2018). Coumarin compounds in medicinal chemistry: some important examples from the last years. Curr. Top. Med. Chem. 18, 124–148. 10.2174/1568026618666180329115523 29595110

[B35] Pignata S. Lorusso D. Scambia G. Sambataro D. Tamberi S. Cinieri S.. (2015). Pazopanib plus weekly paclitaxel versus weekly paclitaxel alone for platinum-resistant or platinum-refractory advanced ovarian cancer (MITO 11): a randomised, open-label, phase 2 trial. Lancet Oncol. 16, 561–568. 10.1016/s1470-2045(15)70115-4 25882986

[B36] Pu W. Li J. Zheng Y. Shen X. Fan X. Zhou J. K.. (2018). Targeting Pin1 by inhibitor API-1 regulates microRNA biogenesis and suppresses hepatocellular carcinoma development. Hepatology 68, 547–560. 10.1002/hep.29819 29381806

[B37] Pu W. Lin Y. Zhang J. Wang F. Wang C. Zhang G. (2014a). 3-Arylcoumarins: synthesis and potent anti-inflammatory activity. Bioorg. Med. Chem. Lett. 24, 5432–5434. 10.1016/j.bmcl.2014.10.033 25453803

[B38] Pu W. C. Mu G. M. Zhang G. L. Wang C. (2014b). Copper-catalyzed decarboxylative intramolecular C–O coupling: synthesis of 2-arylbenzofuran from 3-arylcoumarin. RSC Adv. 4, 903–906. 10.1039/c3ra46414h

[B39] Rao H. S. Sivakumar S. (2006). Condensation of alpha-aroylketene dithioacetals and 2-hydroxyarylaldehydes results in facile synthesis of a combinatorial library of 3-aroylcoumarins. J. Org. Chem. 71, 8715–8723. 10.1021/jo061372e 17080998

[B40] Rovina D. Fontana L. Monti L. Novielli C. Panini N. Sirchia S. M.. (2014). Microtubule-associated protein/microtubule affinity-regulating kinase 4 (MARK4) plays a role in cell cycle progression and cytoskeletal dynamics. Eur. J. Cell Biol. 93, 355–365. 10.1016/j.ejcb.2014.07.004 25123532

[B41] Sack J. S. Gao M. Kiefer S. E. Myers J. E. Jr. Newitt J. A. Wu S.. (2016). Crystal structure of microtubule affinity-regulating kinase 4 catalytic domain in complex with a pyrazolopyrimidine inhibitor. Acta Crystallogr. F Struct. Biol. Commun. 72(Pt 2), 129–134. 10.1107/s2053230x15024747 26841763 PMC4741193

[B42] Schmid P. Adams S. Rugo H. S. Schneeweiss A. Barrios C. H. Iwata H.. (2018). Atezolizumab and nab-paclitaxel in advanced triple-negative breast cancer. N. Engl. J. Med. 379, 2108–2121. 10.1056/NEJMoa1809615 30345906

[B43] Serra S. Chicca A. Delogu G. Vázquez-Rodríguez S. Santana L. Uriarte E.. (2012). Synthesis and cytotoxic activity of non-naturally substituted 4-oxycoumarin derivatives. Bioorg. Med. Chem. Lett. 22, 5791–5794. 10.1016/j.bmcl.2012.07.099 22901895

[B44] Smitha G. Sanjeeva Reddy Ch. (2004). ZrCl4-catalyzed pechmann reaction: synthesis of coumarins under solvent-free conditions. Synth. Commun. 34, 3997–4003. 10.1081/SCC.200034821

[B45] Sugino T. Tanaka K. (2001). Solvent-free coumarin synthesis. Chem. Lett. 30, 110–111. 10.1246/cl.2001.110

[B46] Sun H. L. Cui R. Zhou J. Teng K. Y. Hsiao Y. H. Nakanishi K.. (2016). ERK activation globally downregulates mirnas through phosphorylating exportin-5. Cancer Cell 30, 723–736. 10.1016/j.ccell.2016.10.001 27846390 PMC5127275

[B47] Sun J. Ding W. X. Zhang K. Y. Zou Y. (2011). Efficient synthesis and biological evaluation of 4-arylcoumarin derivatives. Chin. Chem. Lett. 22, 667–670. 10.1016/j.cclet.2010.12.017

[B48] Takaishi K. Izumi M. Baba N. Kawazu K. Nakajima S. (2008). Synthesis and biological evaluation of alkoxycoumarins as novel nematicidal constituents. Bioorg. Med. Chem. Lett. 18, 5614–5617. 10.1016/j.bmcl.2008.08.102 18793855

[B49] Trinczek B. Brajenovic M. Ebneth A. Drewes G. (2004). MARK4 is a novel microtubule-associated proteins/microtubule affinity-regulating kinase that binds to the cellular microtubule network and to centrosomes. J. Biol. Chem. 279, 5915–5923. 10.1074/jbc.M304528200 14594945

[B50] Venkata Sairam K. Gurupadayya B. M. Chandan R. S. Nagesha D. K. Vishwanathan B. (2016). A review on chemical profile of coumarins and their therapeutic role in the treatment of cancer. Curr. Drug Deliv. 13, 186–201. 10.2174/1567201812666150702102800 26135671

[B51] Wang Y. F. Shi Q. W. Dong M. Kiyota H. Gu Y. C. Cong B. (2011). Natural taxanes: developments since 1828. Chem. Rev. 111, 7652–7709. 10.1021/cr100147u 21970550

[B52] Wei J. Wang P. C. Jia Q. F. Huang J. Y. Du Z. Y. Zhang K. (2013). Amine-catalyzed cascade synthesis of 3,4-diunsubstituted coumarins. Euro. J. Org. Chem. 2013, 4499–4502. 10.1002/ejoc.201300538

[B53] Yan W. Yang T. Yang J. Wang T. Yu Y. Wang Y.. (2018). SKLB060 reversibly binds to colchicine site of tubulin and possesses efficacy in multidrug-resistant cell lines. Cell. Physiol. Biochem. 47, 489–504. 10.1159/000489983 29794416

[B54] Zhang Y. Zou B. Chen Z. Pan Y. Wang H. Liang H.. (2011). Synthesis and antioxidant activities of novel 4-Schiff base-7-benzyloxy-coumarin derivatives. Bioorg. Med. Chem. Lett. 21, 6811–6815. 10.1016/j.bmcl.2011.09.029 21978674

[B55] Zheng Y. Pu W. Li J. Shen X. Zhou Q. Fan X.. (2019). Discovery of a prenylated flavonol derivative as a Pin1 inhibitor to suppress hepatocellular carcinoma by modulating microRNA biogenesis. Chem. Asian J. 14, 130–134. 10.1002/asia.201801461 30474357

