# Supplementary material for: Discovery of Coumarin as Microtubule Affinity-Regulating Kinase 4 Inhibitor That Sensitize Hepatocellular Carcinoma to Paclitaxel
Source: Front Chem. 2019 May 24;7:366. doi: 10.3389/fchem.2019.00366 (PMC6543911; doi:10.3389/fchem.2019.00366)

Supplementary Material

Discovery of coumarin as microtubule affinity-regulating kinase 4 inhibitor that sensitize hepatocellular carcinoma to paclitaxel

Xianyan Shen, Xuesha Liu, Shunli Wan, Xin Fan, Huaiyu He, Rong Wei, Wenchen Pu, Yong Peng, and Chun Wang^*^

* Correspondence:
[wangchun@cib.ac.cn](mailto:wangchun@cib.ac.cn)

**Spectra Data:**

**(Z)-3-(2-(Tert-butylthio)phenyl)-2-(4-methoxyphenyl)acrylonitrile (p66)**

**
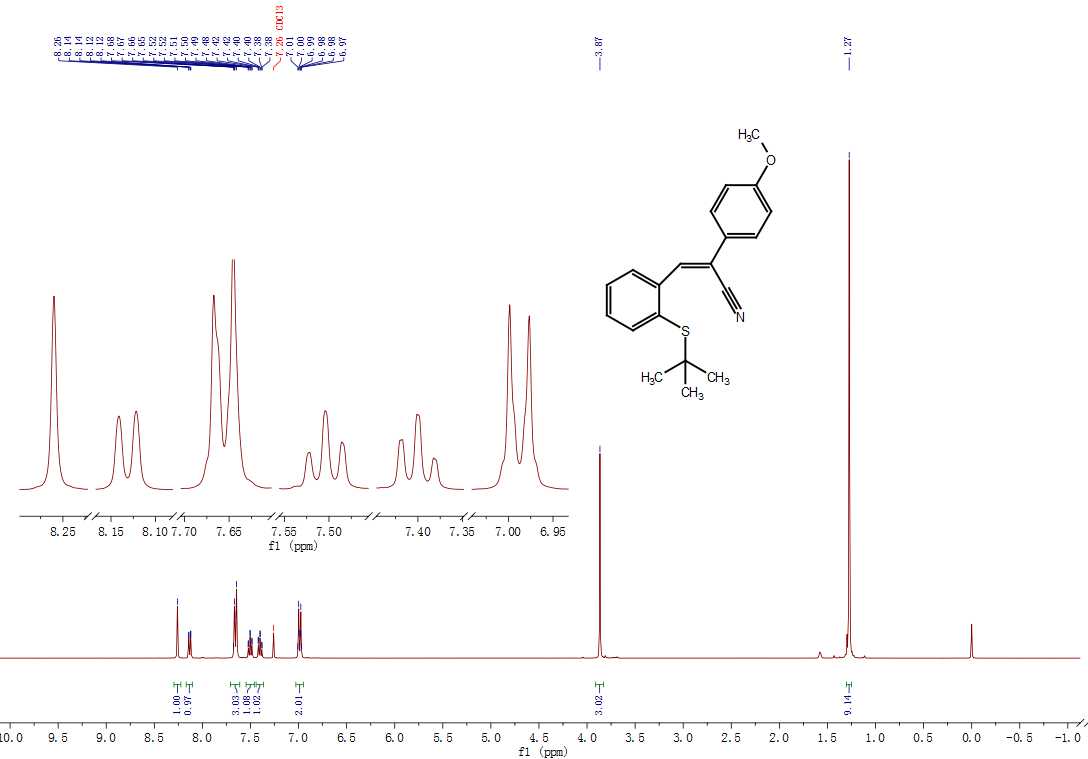
**

**
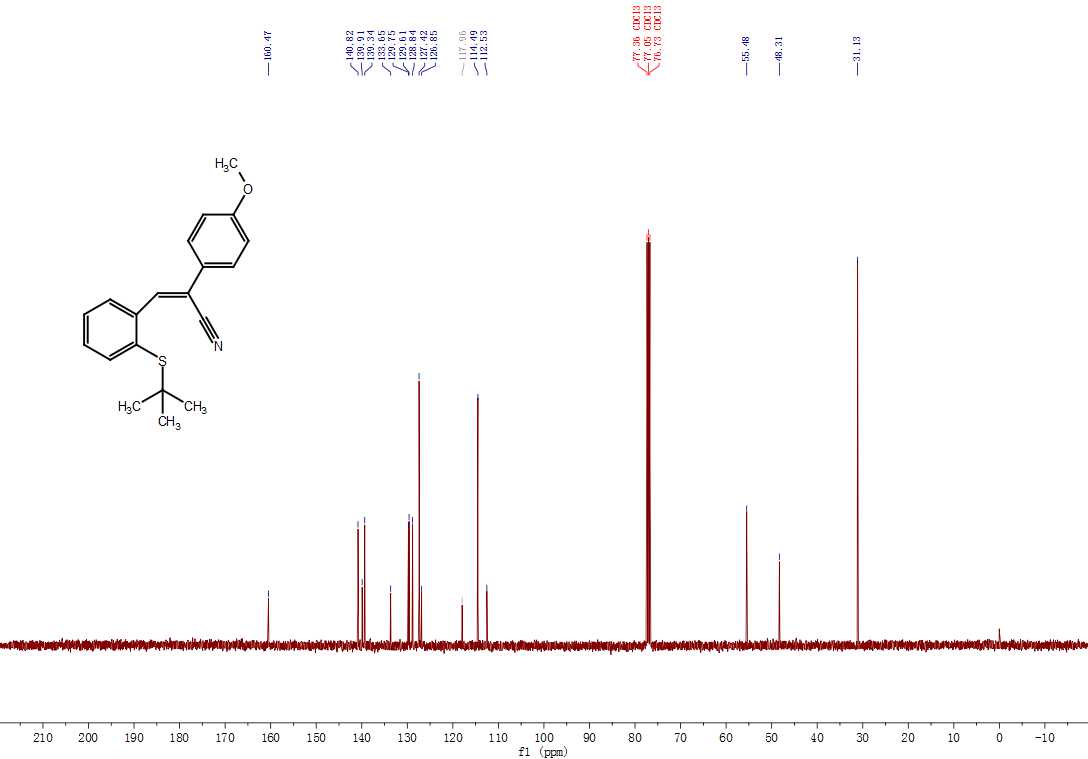
**

**(Z)-3-(2-(Tert-butylthio)phenyl)-2-(3,4-dimethoxyphenyl)acrylonitrile (p67)**

**
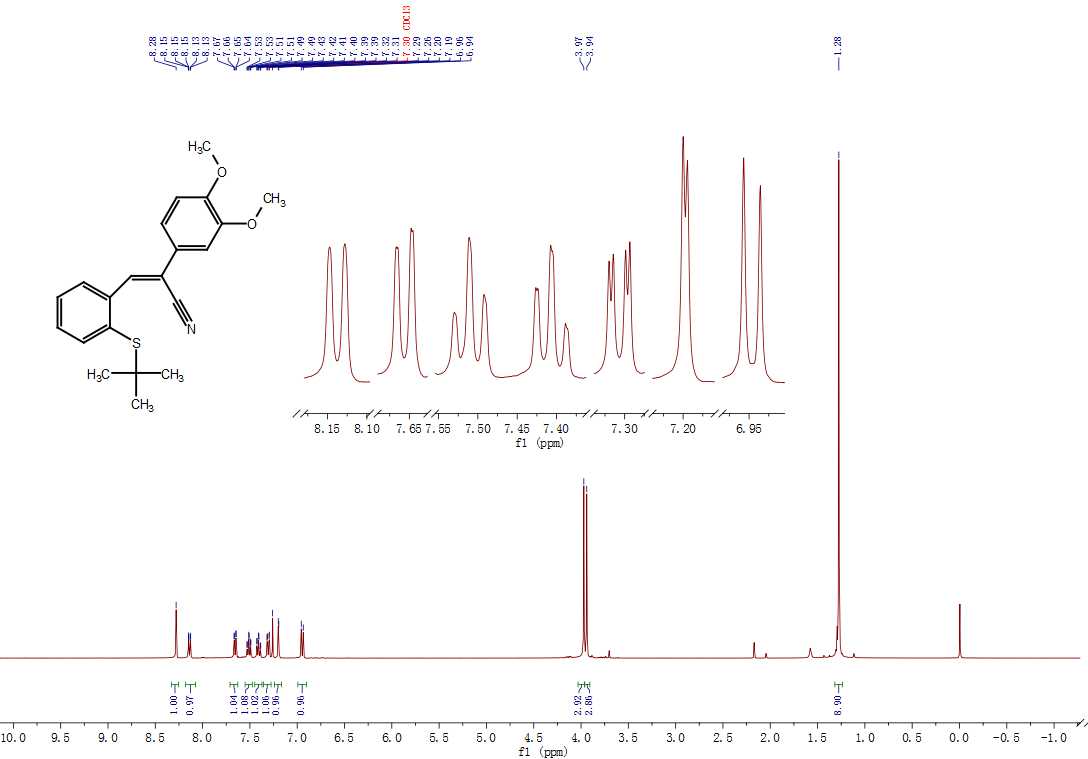
**

**
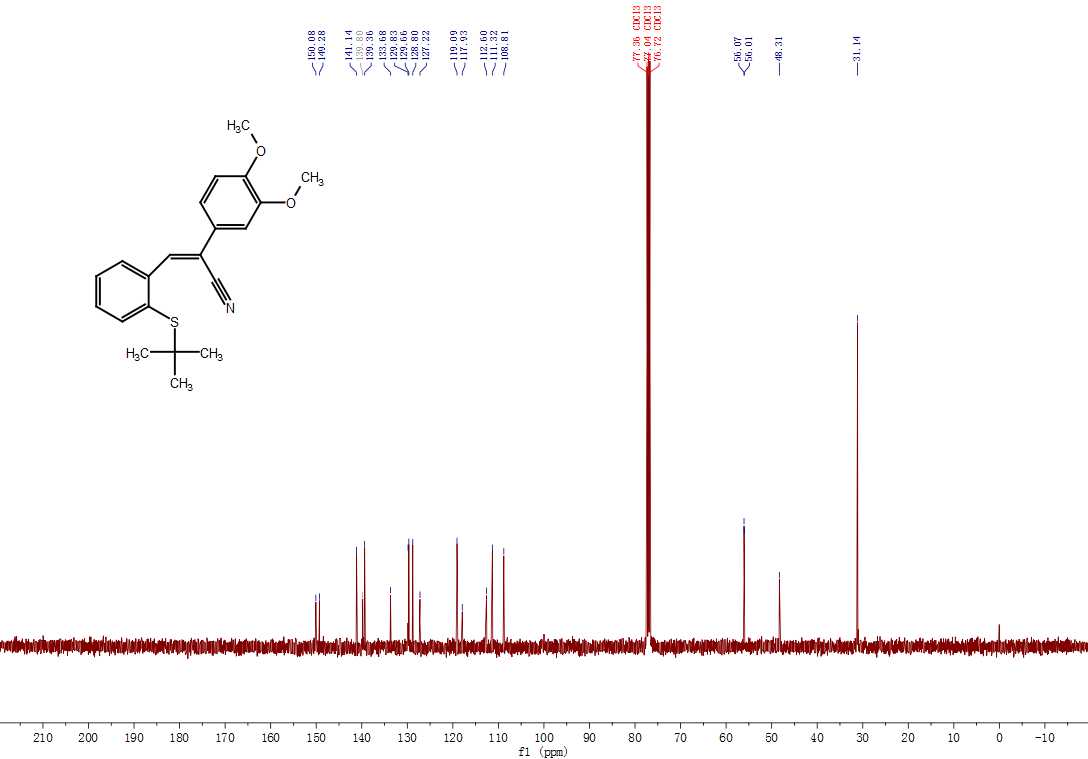
**

**(Z)-3-(2-(Tert-butylthio)phenyl)-2-(4-fluorophenyl)acrylonitrile (p68)**

**
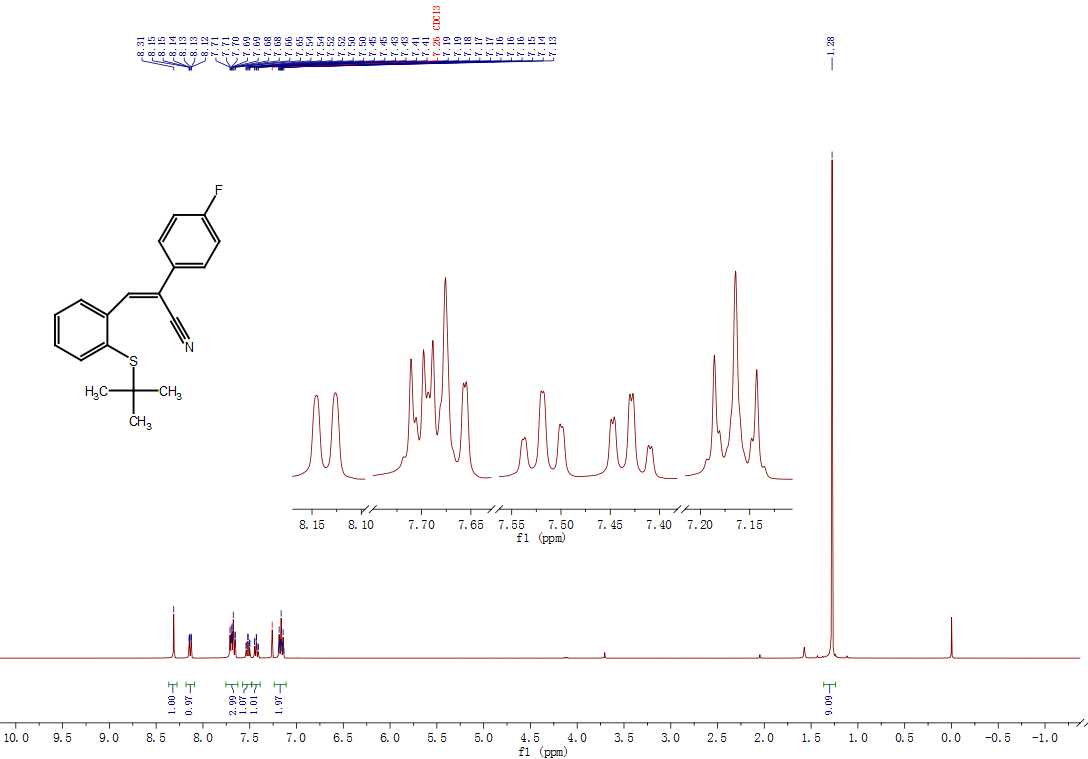
**

**
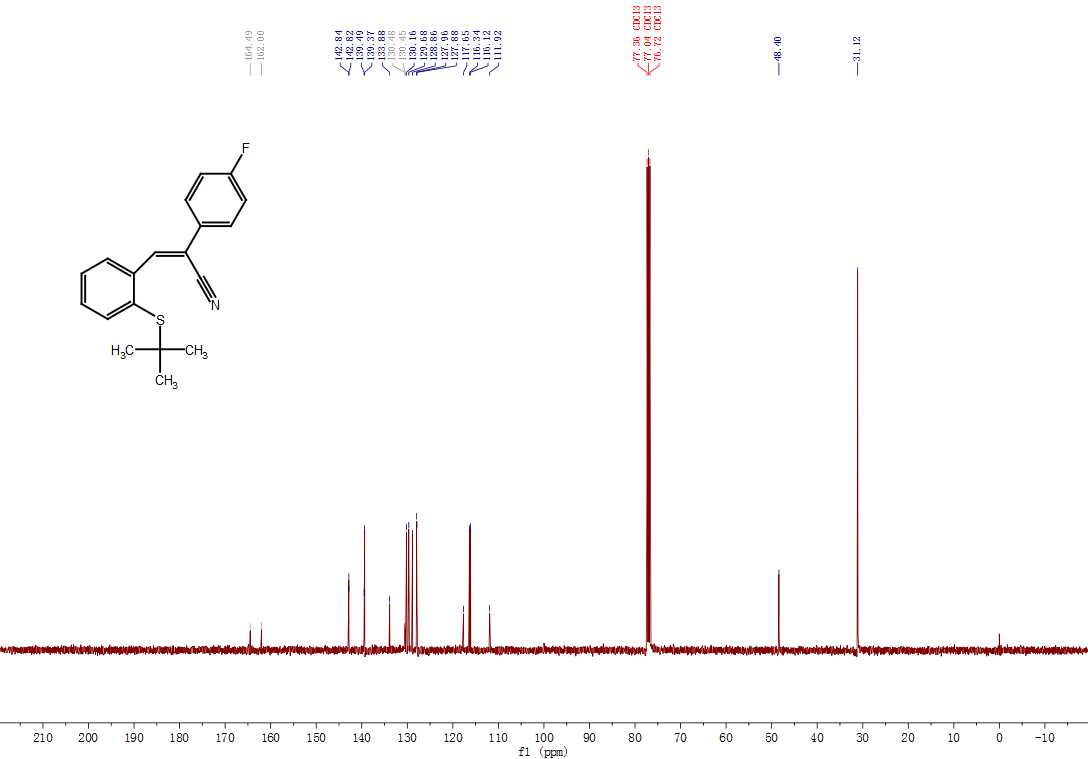
**

**3-(4-Methoxyphenyl)-2H-thiochromen-2-one (66)**


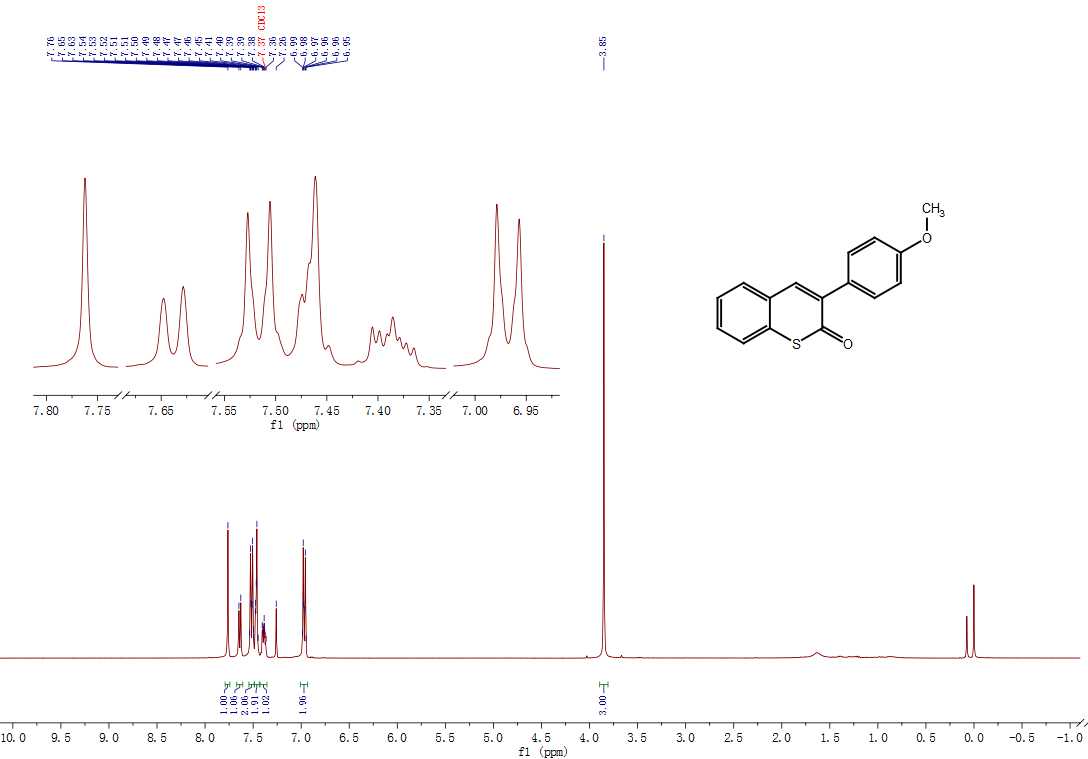

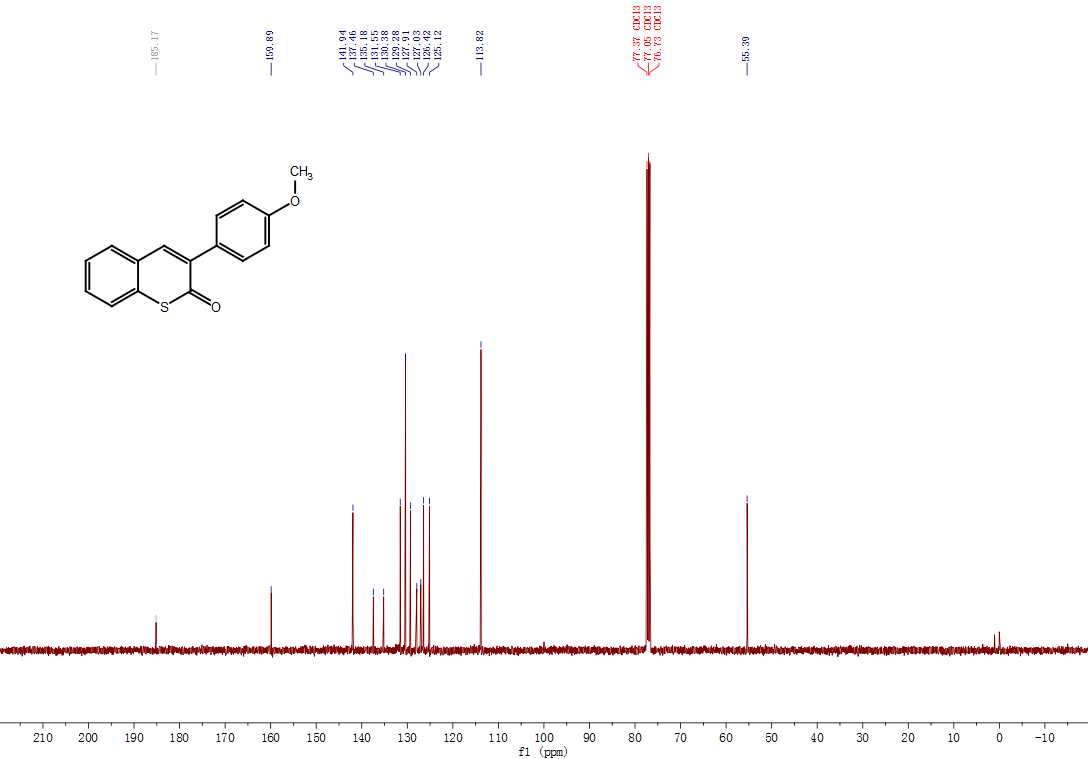


**3-(3,4-Dimethoxyphenyl)-2H-thiochromen-2-one (67)**

**
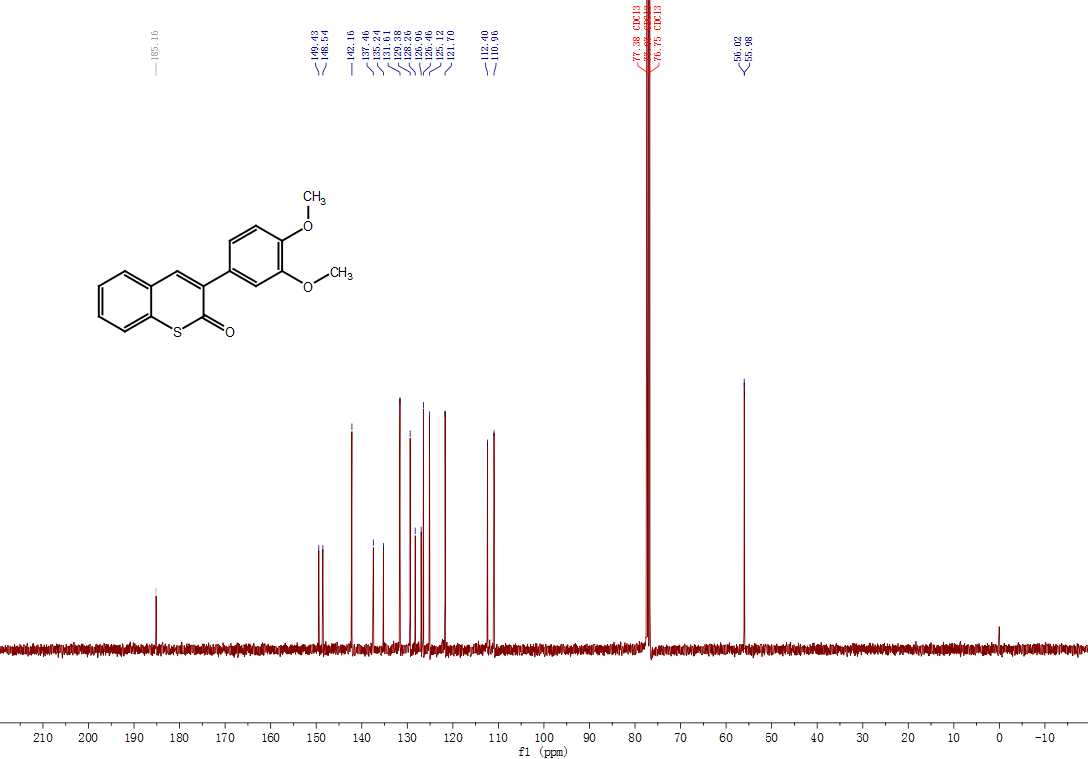
**
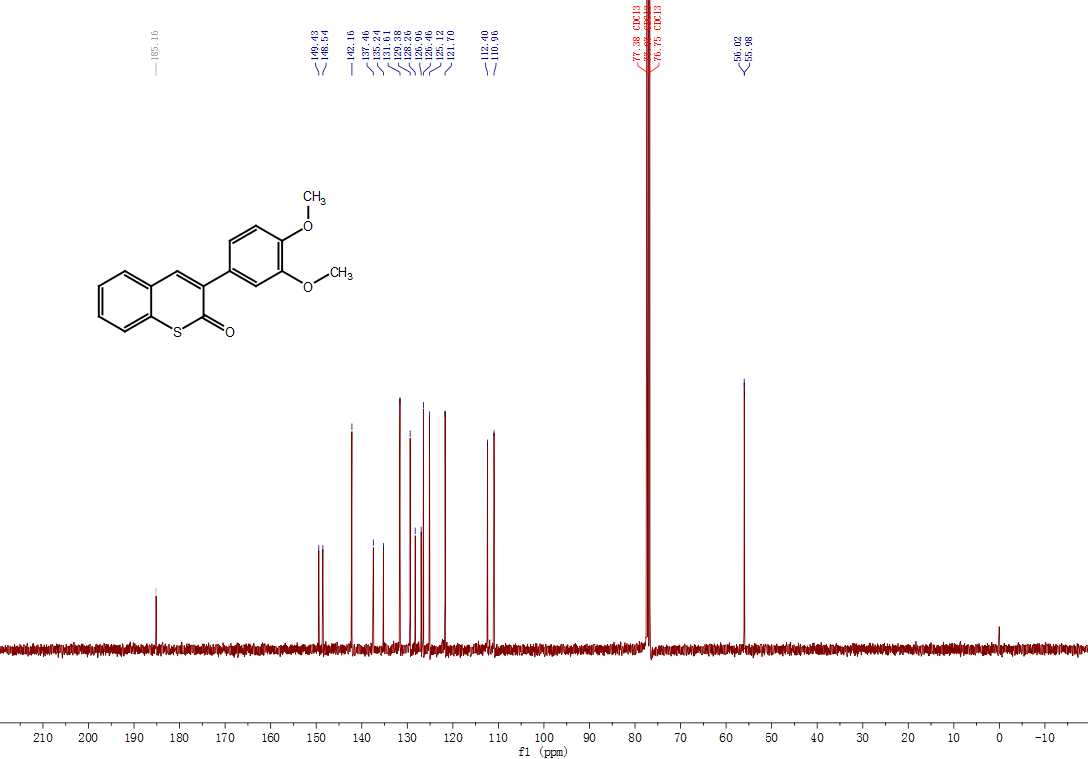


**3-(4-Fluorophenyl)-2H-thiochromen-2-one (68)**


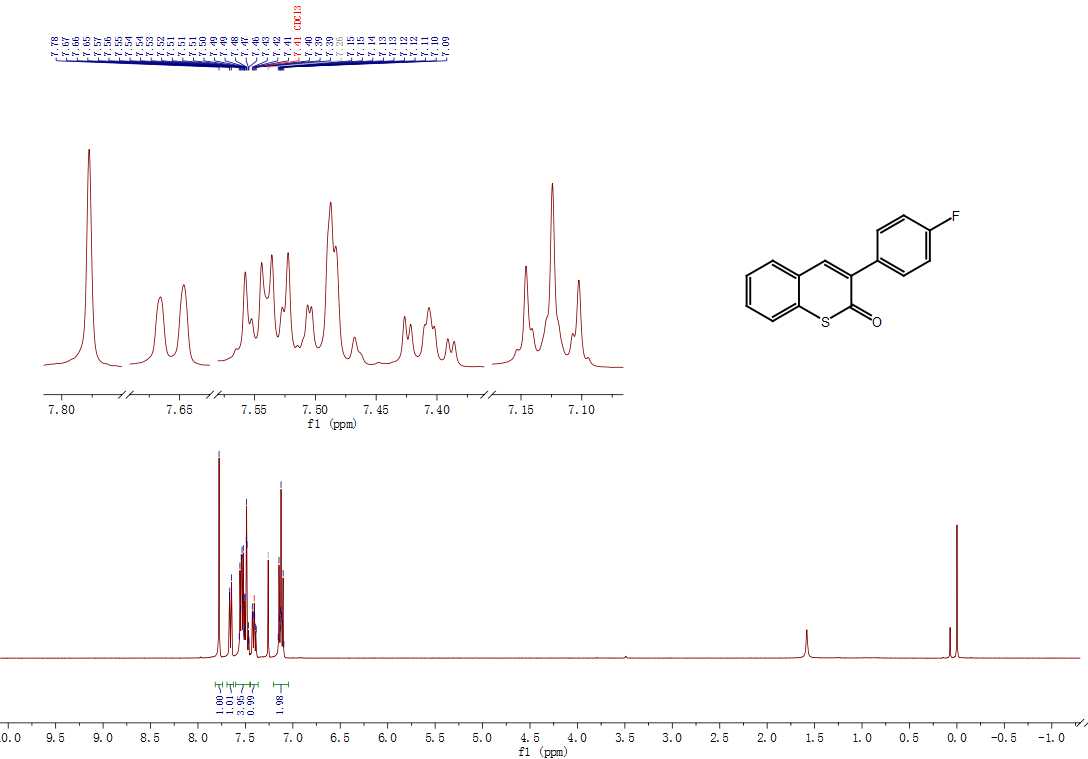

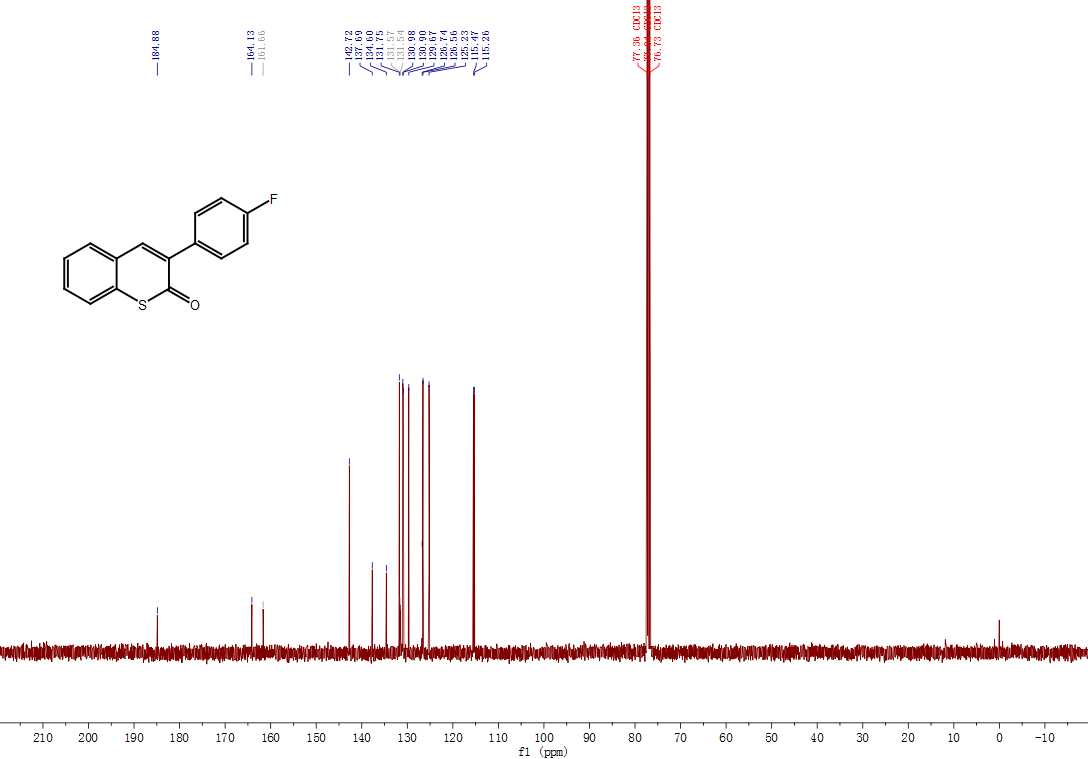

Supplement: Supplementary file 1 [file Table_1.DOCX]
